# Clinical application of single‐cell RNA sequencing in disease and therapy

**DOI:** 10.1002/ctm2.70512

**Published:** 2025-10-31

**Authors:** Aisha Shigna Nadukkandy, Sowmiya Kalaiselvan, Lin Lin, Yonglun Luo

**Affiliations:** ^1^ Department of Biomedicine Aarhus University Aarhus Denmark; ^2^ Steno Diabetes Center Aarhus Aarhus University Hospital Aarhus Denmark

**Keywords:** single‐cell RNA sequencing, the dawn of a new genome medicine era

## Abstract

**Background:**

The emergence of single‐cell RNA sequencing (scRNA‐seq) technology has revolutionized our capacity to study cell functions in complex tissue microenvironments. Traditional transcriptomic approaches, such as microarrays and bulk RNA sequencing, lacked the resolution to distinguish signals from heterogeneous cell populations or rare cell types, limiting their clinical utility. Since 2009, scRNA‐seq has evolved as a new and powerful tool for revisiting somatic evolution and functions under physiological or pathological conditions.

**Main Topics Covered:**

This review focus on elaborating on the clinical applications of scRNA‐seq technology, with a particular emphasis on the application of scRNA‐seq methods in revisiting the somatic cell evolution in human diseases. We further provide a snapshot of the scRNA‐seq applications in biomarker discovery and drug development, current challenges associated with the technology, and future directions.

**Conclusions:**

With the recent progresses in single cell and spatial transcriptome technologies, scRNA‐seq enables a deeper understanding of the complexity of human diseases. The integration of AI and machine learning algorithms into big data analysis offers hope for overcoming these hurdles, potentially allowing scRNA‐seq and multi‐omics approaches to bridge the gap in our understanding of complex biological systems and advances the development of precision medicine.

**Highlights:**

- This review provides a systematic overview of the application of scRNA‐seq technology in understanding of disease mechanisms.
- We cover applications in respiratory diseases, metabolic disorders, cardiovascular diseases, cancers, autoimmune and auto‐inflammatory diseases, neurodegenerative diseases, and infectious diseases.
- This review also explores promises and challenges for the emerging application of scRNA‐seq in drug discovery.

## Graphical abstract

This review provides a comprehensive overview of the application of scRNA‐seq in revisiting the somatic cell evolutions in human diseases, as well as in biomarker discovery and drug development.

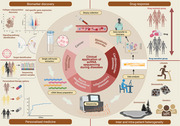

## 1 INTRODUCTION

**FIGURE 1:**
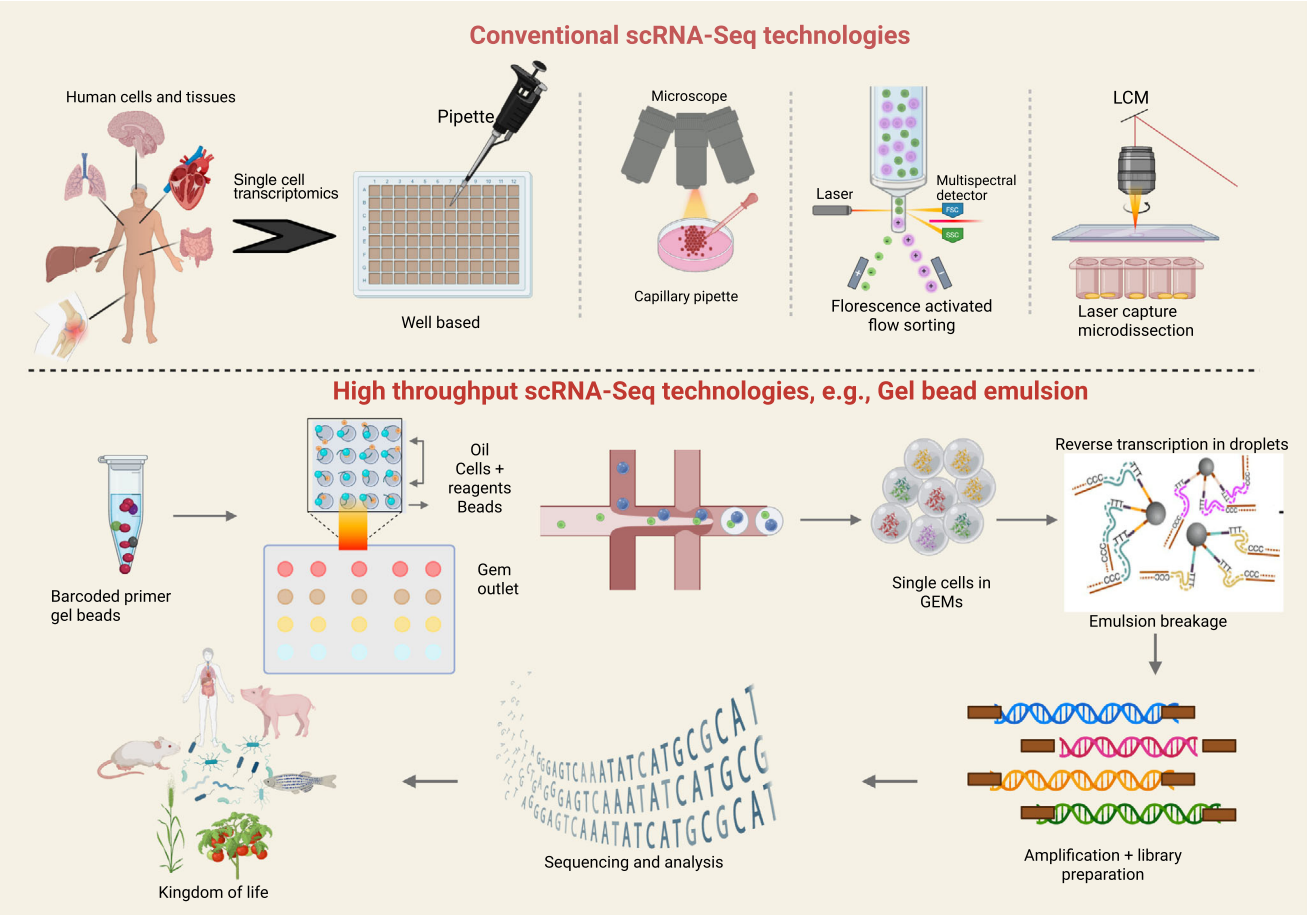
Schematic diagram depicting the difference between the conventional and microfluidic‐based scRNA‐seq technologies. In conventional techniques, cells are isolated using micropipettes, fluorescent‐activated cell sorting system and microdissection using laser capture techniques. Recent developments in microfluidic‐based techniques like droplet, valve and nano‐well has revolutionised the scRNA sequencing, among which the most common is the droplet‐based technique. High‐throughput scRNA‐seq is exemplified with 10× Genomics gel bead in emulsion (GEM) technique which encapsulates barcoded single cells in a hydrogel microsphere where all the reactions are carried out in a droplet to prepare the cells for RNA sequencing. Figure is created with Luo, Y. (2026) https://BioRender.com/ks0ul5p.

In 2009, Tang et al. introduced the conception and technological cornerstone of single‐cell RNA sequencing (scRNA‐seq), which integrated RNA‐seq technology with single‐cell cDNA amplification to investigate mouse embryos during early development. This state‐of‐the‐art study demonstrated that scRNA‐seq technology could detect 75% more genes compared to microarrays in a mouse blastomere and identified 1753 previously unknown splice junctions.^1^ Since its inception, a multitude of scRNA‐seq techniques have been developed (Figure 1), each utilising distinct strategies for cell capture, transcript barcoding and amplification. Variations among these scRNA‐seq methods result in differences in transcript length, target cell number and read depth. Nevertheless, scRNA‐seq protocols typically converge upon a common workflow: commencing with sample preparation and dissociation, followed by single‐cell capture, transcripts barcoding, reverse transcription (RT), cell lysis and cDNA amplification, and culminating in the construction of sequencing libraries and subsequent RNA sequencing.^2^ For more technical detail regarding the scRNA‐seq, we refer readers to previous reviews from us and other groups.^3^, ^4^, ^5^

Accurate sample preparation is crucial for generating high‐quality single‐cell transcriptome data. Given the inherent diversity among cell types, protocols must be diligently optimised to accommodate variables such as cellular dimensions, viability and cultivation conditions.^6^ Typically, single‐cell suspensions are procured through a confluence of enzymatic and mechanical dissociation techniques. Subsequent to dissociation, individual cells are captured after employing an array of methodologies, such as plate‐based fluorescence‐activated cell sorting (FACS) and droplet‐based systems.^7^ For example, the Chromium system by 10× Genomics, a preeminent droplet‐based platform, epitomises a microfluidic innovation that facilitates the rapid, simultaneous profiling of thousands of cells within discrete droplets. However, this system constrains cell diameter to less than 30 µm. For cells exceeding this dimension, plate‐based FACS employing nozzles of up to 130 µm offers a feasible alternative for cell capture. Compared to scRNA‐seq, single‐nuclei RNA sequencing (snRNA‐seq) presents a viable substitute and conventional RNA sequencing at single‐cell levels. Particularly in relation with clinical research and applications, the snRNA‐seq does not require immediate processing of the clinical samples, providing many advantages in term of practicalities. These highly valuable clinical samples could be snap frozen and stored properly at a temperature of approximately 80°C. Through nuclear dissociation techniques, snRNA‐seq proficiently isolates nuclear RNA, thereby mitigating potential technical complications and batch effects.^8^ For both scRNA‐seq and snRNA‐seq, upon cell capture, all transcripts from individual cells are barcoded, reverse transcribed into barcoded cDNA, followed by second‐strand synthesis and polymerase chain reaction (PCR)‐based cDNA amplification. For simplicity, we refer both scRNA‐seq and snRNA‐seq as ‘scRNA‐seq’ unless specified. The droplet‐based systems employ pooled PCR coupled with cell barcoding techniques, markedly enhances throughput.^9^ Post‐cDNA amplification, deep sequencing libraries are constructed from the barcoded cDNA and sequenced utilising high‐throughput next‐generation sequencers. Deep sequencing libraries constructed with 3′ end enrichment are cost effective and produce reduced sequencing noise, while libraries retaining full‐length transcripts typically offer superior transcriptome insights, such as alternative splicing and isoforms.^10^ Thus, the diverse array of available scRNA‐seq methodologies presents a considerable opportunity but also a challenge in selecting the most appropriate platform for a study. The selection of a platform is profoundly influenced by the specific research inquiry, the nature of the biological sample and the available financial resources. To date, numerous protocols and scRNA‐seq methodologies and platforms, each varying in sensitivity, throughput and cost, have been exhaustively reviewed in the literature.^4^, ^11^, ^12^ While an in‐depth examination of these technologies is beyond the focus of this review, it is crucial to underscore their main aspects and limitations to foster further discourse on their application in elucidating various disease pathologies and mechanisms.

With the maturation of scRNA‐seq methods, the analysis, interpretation and presentation of the massive data generated are regarded as the most important and the time consuming steps. Few, if any, comprehensive ‘plug‐and‐play’ solutions exist for the quality control (QC), preliminary analysis and interpretation of scRNA‐seq data. Nevertheless, companies specialising in wet‐lab hardware and reagents for scRNA‐seq, such as 10× Genomics with its Loupe software and Fluidigm with Singular, are progressively providing complementary software to support these needs. While these tools are designed to be user‐friendly, they often lack details concerning their specific algorithmic methodologies and parameters, particularly for further data integration and analyses. Despite ongoing advancements, a definitive gold‐standard analytical platform remains elusive in this domain. Recent reports suggest that more intuitive, web‐browser‐based interfaces will soon become available,^13^ though the exact features are still under development. Thus, specialised bioinformatic support remains indispensable for biomedical researchers and clinicians engaged with scRNA‐seq data. Prior to initiating any analysis, scRNA‐seq data undergo a series of bioinformatic QC procedures. These checks are critical for excluding subpar data from individual cells, which may arise from factors such as compromised cell viability at the time of lysis, inefficient mRNA recovery or inadequate cDNA synthesis. While a universally accepted filtering strategy remains elusive, standard QC criteria encompass the evaluation of relative library size, the number of detected genes, and the proportion of reads aligning with mitochondrial genes or synthetic spike‐in RNAs.^14^ Recently, more sophisticated methods have been developed for identifying low‐quality cells.^5^ It is also prudent to consider the possibility of multiple cells being inadvertently included in a single assessed sample, alongside verifying successful single‐cell isolation. The ability to assess contamination at the point of single‐cell isolation varies according to the employed technique. Once scRNA‐seq data have been purged of suboptimal samples, principal component analysis (PCA) is commonly employed for dimensionality reduction, serving as a crucial instrument for probing the heterogeneity within the scRNA‐seq dataset. This analysis is frequently augmented by advanced machine learning algorithms, such as t‐distributed stochastic neighbour embedding (t‐SNE) and Gaussian process latent variable modelling (GPLVM), which have been comprehensively reviewed in the literature before.^4^ Following dimensionality reduction and visualisation, cells are often categorised into subpopulations based on their transcriptome profiles that often reveal biologically significant patterns, such as functional similarities or developmental trajectories. Given the high dimensionality of scRNA‐seq data, numerous specialised methodologies have been developed to address this complexity.^15^, ^16^ An increasing array of algorithms and computational methodologies are being developed to assist researchers in deciphering molecular relationships between single cells as characterised by scRNA‐seq, thereby enhancing the insights gleaned from mere clustering. These trajectory‐inference methodologies are predicated on the identification of intermediate cellular states, with the most advanced tools now capable of tracing both linear differentiation pathways and multifaceted fate decisions.^17^, ^18^ Although these approaches currently necessitate at least rudimentary programming expertise, the source codes for these methodologies are typically available for bioinformaticians to access and utilise. Effective analysis of scRNA‐seq data thus demands a robust collaborative relationship with bioinformaticians. Particularly noted two of these resources, the SEURAT (https://satijalab.org/seurat/) and the Galaxy Europe Single Cell Lab (https://singlecell.usegalaxy.eu) are hallmark examples that provide highly valuables bioinformatic tools and resources for analysing these scRNA‐seq data.

As research in scRNA‐seq progresses, the capabilities of these methods are continually advancing, leading to lower detection costs and enhanced insights into molecular mechanisms at the single‐cell level. For instance, the method single‐cell combinatorial indexed sequencing (SCI‐seq), suggested by Vitak et al. can construct numerous single‐cell libraries and simultaneously detect somatic cell copy number variations.^19^ This technique, which enhances cell detection while lowering library construction costs, is significant for somatic cell variation investigations. A unique single‐cell whole‐genome amplification technique was developed by Chen et al. that can more accurately discover mutations in a wider range of illnesses and detect copy number variants (CNVs) at kilobase resolution.^20^ A single‐cell multiple sequencing method (scCOOL‐seq) was developed by Guo et al. to analyse single‐cell chromatin state/nuclear niche localisation, copy number variations, ploidy and DNA methylation simultaneously. These results can reveal various patterns and functions of chromatin state and DNA methylation.^21^ Topographic single‐cell sequencing (TSCS), developed by Casasent et al. gives precise spatial position data for individual cells.^22^ This technique precisely quantifies and characterises the unique attributes of individual tumour cells in space, enabling an in‐depth investigation into tumour cell invasion and metastasis. Demaree et al. developed a high‐yield, precise single‐cell sequencing (SiC‐seq) method that individuates, amplifies and barcodes individual cells via droplet microfluidics.^23^ This method expands the scope of genomic research for various cell types. The Microwell‐seq developed by Han et al. is a high‐throughput and low‐cost scRNA‐seq platform.^24^ Not only does it improve the detection abundance of single‐cell technologies, but it also reduces the cost of detection by an order of magnitude compared to single‐cell sequencing techniques coated with oil droplets. The SPLit‐seq technology from Rosenberg et al., based on the principle of a low‐cost combined barcode, can reduce the cost of single‐cell transcriptome sequencing to 1 cent, once again breaking the cost threshold for single‐cell detection.^25^ More recently, based on high‐density DNA nanoball‐patterned arrays, Stereo‐cell developed by Liao et al., provides a high resolutions method for studying individual cells’ transcriptomes at spatial levels, and most importantly compatible for a wide range of cell numbers and sizes. Building on the trajectory of scRNA‐seq, we foresee a continuous development and revolution in the scale, robustness, sensitivity, broadness and compatibility. Particularly, the implementation of large scRNA‐seq data and deep‐learning algorithms (or artificial intelligence) will transform single‐cell multi‐omics research and its applications in biomedical, clinical and pharmaceutical investigations.

### 1.1 Single cell transcriptomics in disease characterisation and biomarker identification

**FIGURE 2:**
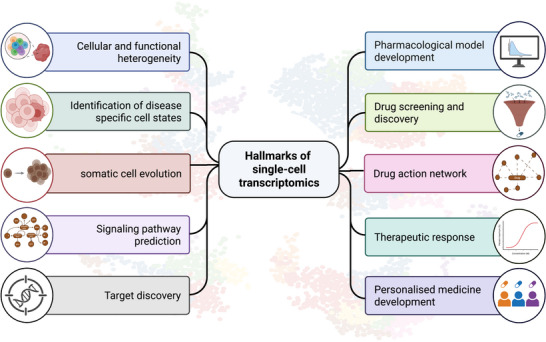
Schematic representation of the applicational hallmarks of single‐cell transcriptomics in disease understanding and drug discovery. This figure is created in BioRender. Luo, Y. (2026) https://BioRender.com/ks0ul5p.

**FIGURE 3:**
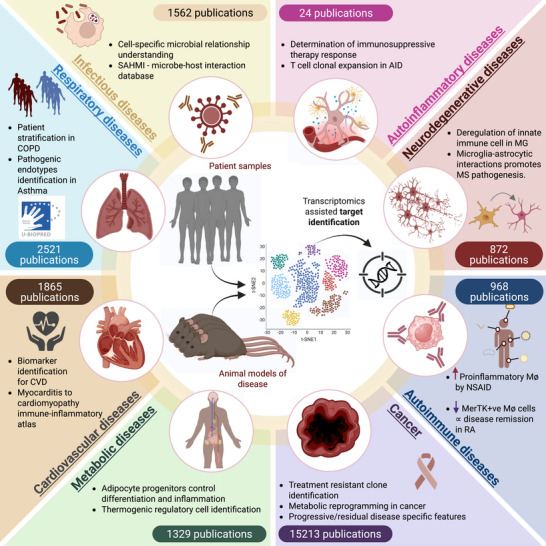
Schematic representation of how single‐cell transcriptomics have revolutionised the studies of various diseases and their respective drug discovery. The number of publications available on PubMed for the respective diseases (statistics extracted using R studio) are also highlighted. This figure is created in BioRender. Luo, Y. (2026) https://BioRender.com/4clk61m.

Previously, studies on gene expression and transcription activities in cells/tissues were mainly depended on the techniques like microarrays, quantitative PCR (qPCR) and bulk RNA sequencing. The need for a substantial amount of starting materials and the inability to distinguish the heterogeneous nature of the cellular composition of the tissue being investigated made the process of understanding disease initiation and progression highly inefficient. Even though techniques like FACS, immunohistochemistry and cytometry by time of flight (CyTOF) that could profile cellular differences at single‐cell level, the need for prior insights on the testable targets and limited genes detected represent a limit for these single‐cell profiling methods.^26^ Thus, the constant search for innovative tools that could help accelerate effective biomarker identification and treatment development persisted for decades. The entry of single‐cell sequencing technologies improved the understanding of diseased cell states and altered cell composition in patient samples and relevant preclinical models, which pave way towards the identification of novel molecular targets for clinical applications. Particularly, rare and disease‐associated cell types are frequently not detected by bulk RNA sequencing. Furthermore, most differentially expressed genes detected by bulk RNA sequencing are not transcriptomically up‐regulated. For instance, most immune‐related genes were found to be up‐regulated in diseased samples, such as livers of metabolic dysfunction‐associated steatohepatitis (MASH), as revealed by bulk RNA sequencing.^27^ However, the causes of these elevated expression of immune‐related genes in the MASH livers could be due to transcription activation and/or infiltration of immune cells. The process of drug candidate selection has also been accelerated due to additional insights on the off‐target effects, heterogeneous patient responses and elucidation of the mechanism of drug action, as well as resistance^26^ (Figure 2). Here we will be discussing published studies that demonstrate the potential of sc‐RNA seq technologies in understanding the diseases and in biomarker discovery for various diseases at different stages of life. A summary of the findings are also included as an illustration (Figure 3).

## 2 RESPIRATORY DISEASES

Although the genetic information in a human body is identical regardless of the tissue it comes from, the transcriptome varies drastically between organs and even the cells within them.^28^ These dynamic changes can occur due to environmental exposures, disease conditions or senescence. This has especially perplexed the community of respiratory researchers due to the highly complex cellular heterogeneity of the respiratory system as a whole.^29^ Single‐cell technologies has greatly broadened the knowledge on the development of respiratory system, heterogeneity of cell types within the human lungs in both healthy and disease conditions, which in turn enables a better understanding of patient characterisation, environmental exposure and intervention causal effect, and drug response.^30^, ^31^, ^32^, ^33^ This has been achieved in various respiratory diseases like asthma, chronic obstructive pulmonary disease (COPD), idiopathic pulmonary fibrosis (IPF) and pulmonary arterial hypertension (PAH).

Asthma is a multifaceted chronic respiratory disease known to affect 300 million individuals globally.^34^ With diverse clinical phenotypes and pathogenic endotypes, asthma patients present with various clinical symptoms like coughing, wheezing and dyspnoea caused as a result of airway inflammation and hyperresponsiveness. Even though the combination of steroids with agonists to beta2‐adrenergic receptors exist as a treatment for asthma, poor response or tolerance to beta2‐agonists and glucocorticoids is a significant cause of concern for patients as a consequence of complex pathogenesis, disease heterogeneity or individual differences. Recently, multiple single‐cell transcriptomic studies have been conducted on blood cells, airway epithelial cells and sputum of asthma patients, providing many new insights into the disease mechanism and disease heterogeneity.^35^ Studies on peripheral blood mononuclear cell (PBMCs) of severe asthmatic and healthy controls using scRNA‐seq distinguished several immune cells like CD4+ T cells, CD8+ T cells, NK cells and B cells of severe asthmatics express pro‐inflammatory genes such as *JAK1*, *NEAT1* and *IL32*.^36^ Several pro‐inflammatory and remodelling genes like *NOS2*, *CEACAM5* and *CST1* were also transcriptomically up‐regulated in goblet cells of chronic childhood asthma patients compared to healthy.^33^ This study also identified an increase in mast cells in asthmatic patients. Additionally, an increase in the number of monocytes, CD8+ T cells and macrophages was also observed in bronchoalveolar lavage fluids of patient samples.^37^ Large‐scale projects like the Unbiased Biomarkers in Prediction of Respiratory Disease Outcomes (U‐BIOPRED) consortium have also identified the pathogenic endotypes of asthma with the help of gene expression profile‐based analytical methods.^38^ The Severe Asthma Research Program (SARP) has also distinguished asthma endotypes via a weighted gene co‐expression network analysis because the genes associated with epithelial growth and repair and neuronal function were decreased in sample with higher severity.^39^

Like asthma, COPD is a disorder that exhibits heterogenous endophenotypes driven by several molecular networks and signalling pathways.^40^ Often, COPD patients presents with airway limitation and chronic inflammations of the lungs, which is treated using bronchodilators. Application of scRNA‐seq technologies on COPD patient samples provided new insights on the cellular composition of the respiratory tract during the disease. Cells like monocytes, mast cells and AT2 cells are associated with the pathology and their transcriptomic changes indicated mitochondrial dysfunction and immune response.^41^ A study of PBMCs of past and current smokers with and without COPD and emphysema identified 26 genes representing immune, inflammatory responses and sphingolipid metabolism when compared to healthy samples.^42^ Other next‐generation sequencing (NGS) studies have also helped develop prospective patient stratification methods by identifying high‐risk COPD and poorer outcomes.^43^, ^44^ Dominance of neutrophilic‐*Heamophilus* and *Prevotella* airway microbiomes are correlated with poor prognosis and better clinical outcome, respectively.^45^, ^46^ Thus, other than gene therapy, microbiome‐based therapy should also be one of the major strategies put forward in accelerating clinical practise.

Studies that compared transcriptomic changes in COPD and IPF identified an increase in the expression of genes involved in the p53/hypoxia pathway when compared to healthy subjects, which reflects overlapping biological processes between the two respiratory diseases.^47^ IPF is an ultimately lethal and chronic lung disease which are treated using antifibrotic agents that help in slowing down disease progression, which is a ‘one‐size‐fits‐all’ approach disregarding severity, inter and individual molecular and genetic heterogeneity. Several studies have helped identify diagnostic, prognostic and theragnostic biomarker candidates in IPF in recent years.^48^ Expression signatures of 52 genes in the last decade and 13 gene signatures, recently developed using unsupervised clustering, were correlated with a high risk of mortality in peripheral blood of IPF patients.^49^, ^50^ Multi‐omics analysis of the samples available in the Idiopathic Pulmonary Fibrosis Prospective Outcomes (IPF‐PRO) Registry (NCT01915511) registry identified distinct clinical characteristics associated with two novel molecular subtypes of IPF. Between the two, subtype 1 had higher disease severity while enrolling and thus required limited time for disease progression than patients in subtype 2, even after taking into consideration disease severity and antifibrotic treatment.^51^ These findings above could be used as biomarkers for prognostic enrichment during clinical trials.

Though not as common as asthma, COPD and IPF, PAH is a respiratory disorder often seen in humans with elevated pulmonary vascular resistance and subsequent right ventricular failure.^52^ Currently, most of the transcriptomic studies conducted for PAH use RNA from lung homogenate, PBMCs and pulmonary artery smooth muscle cells. It was found that BMP2 and its corresponding receptor, BMPR2, have expression changes associated with PAH. Even though BMPR2 undergoes mutation in most familial PAH, it is known that the gene undergoes differential expression even in patient samples lacking BMPR2 mutation.^53^, ^54^ Other than these, elevated ESR1 gene expression and genes in pathways related to vascular remodelling were also identified in PAH.^55^ Most of the replicable findings from the transcriptomic studies of asthma, COPD, ARDS, IPF and PAH are the subject of ongoing functional validation studies. Combined with data obtained from genomic, epigenomic, metabolomic and proteomic findings, this has identified several targets for respiratory disease‐related prognosis and therapeutics. Sphingolipids are now biomarkers for COPD, and octane and acetaldehyde, in combination with exhaled breath condensate, are imminent biomarkers for ARDS. Studies identifying heterogenous endotypes of asthma, COPD and IPF have thus far shown the potential of omics data‐based classification strategies, which also needs to be extended towards rarer diseases like ARDS and PAH.^56^ To date, the application of scRNA‐seq in respiratory diseases are underrepresented.

## 3 METABOLIC DISORDERS

Metabolic disorder is one of the most prevalent health issues today, affecting nearly 30% of the world's population. It is a major and escalating public health and clinical challenge globally, driven by urbanisation, surplus energy intake, increasing obesity and sedentary lifestyles. This disorder encompasses a group of diseases, including type‐II diabetes, obesity, NAFLD (or MAFLD) and cardiometabolic disorders. Approximately 650 million adults suffer from obesity, a significant cause of numerous fatal conditions like cardiovascular diseases, diabetes and several types of cancers. Usually, when energy intake surpasses energy expenditure, the body typically responds by expanding adipose tissue. This expansion manifests as an increase in both the number and size of adipocytes, along with alterations in the population of adipose vascular stromal cells, the invasion of immune cells and the polarisation of resident cells into a pro‐inflammatory type 1 state. These inflammatory changes lead to metabolic dysfunction, resulting in lipolysis, fibrosis and insulin resistance.^57^ New research utilising scRNA‐seq on human adipose tissues from distinct sites and conditions has yielded notable findings. For examples, using scRNA‐seq, studies have identified novel subpopulations of adipocyte progenitors controlling differentiation and inflammation.^58^, ^59^ Combining scRNA‐seq and cell trajectory analysis, researchers have identified a new subpopulation of adipocyte progenitor cells, the dipeptidyl peptidase 4 positive DPP4^+^ population, predominantly within the mesenchyme.^60^ In addition, a similar approach was employed in a human study where scRNA‐seq cluster analysis of 26 350 cells from 25 fat samples identified 17 distinct cell types, particularly depot and disease‐associated cell types.^61^ Moreover, a subpopulation of cells (CYP2E1^+^) regulating thermogenesis in mouse adipose tissue was identified via scRNA‐seq (snRNA‐seq), and these cells were found to be more common in human adipose tissue.^62^ These findings indicate that targeting these cells could potentially restore thermogenic activity.

Non‐alcoholic fatty liver disease (NAFLD), or recently termed as metabolic dysfunction‐associated fatty liver disease (MAFLD), is a progressive metabolic condition characterised by hepatic lipid accumulation, inflammation, fibrosis and insulin resistance. As the most common form of chronic liver disease, NAFLD encompasses a range of pathological changes from simple steatosis to inflammation, fibrosis and cirrhosis.^63^ Recent scRNA‐seq‐based assays performed on healthy and diseased human liver samples have identified major regulators of cell–cell interactions and genes potentially linked to hepatic fibrosis. By comparing the single‐cell transcriptome in healthy and NAFLD mouse liver, distinct expression profiles of Kupffer cells and monocyte‐derived macrophages during the development of NAFLD were revealed, suggesting that Kupffer cells play a stronger immune role. In contrast, monocyte‐derived macrophages have a pronounced ability to differentiate.^64^ In addition to immune cells, single‐cell transcriptional profiles of hepatocytes from high‐fat diet (HFD)‐induced NAFLD mouse liver showed that HFD triggers substantial transcriptome changes in hepatocytes.^65^ These studies collectively enhance our understanding of cellular heterogeneity and hepatocyte alterations in NAFLD at the single‐cell level, offering valuable insights into the disease's pathogenesis and potential therapeutic targets.^66^, ^67^ Yet, current techniques cannot completely delineate the cellular heterogeneity of the entire liver, thus a recent study applied both scRNA‐seq and snRNA‐seq to identify previously unknown cellular subsets in human liver tissue. This identification was further validated, and its spatial arrangement was identified using immunohistochemistry and spatial transcriptomics.^68^

Like obesity and NAFLD, diabetes remains a significant medical burden worldwide. The increasing recognition of diabetes risk factors and preventive programs are inadequate to stem the global rise in diabetes incidence and prevalence. Lawlor et al. used scRNA‐seq to distinguish pancreatic endocrine and exocrine cells in healthy individuals and T2D patients.^69^ Similarly, Li et al. and Wang et al. used scRNA‐seq to identify various cells in the human pancreas, revealing distinct gene expression patterns in α‐ and β‐cells, which play different roles in the pancreas due to their unique expression of major hormones‐related genes.^70^, ^71^ Interestingly, genes specific to body mass index (BMI)‐associated cell subsets were identified in the subpopulations of endocrine and exocrine cell types. Furthermore, research using Western populations has enriched the available resources.^72^ Dorajoo et al. applied scRNA‐seq to pancreatic islet cells from three non‐diabetic Singaporean Chinese individuals, advancing the understanding of diabetes pathogenesis in East Asian populations.^73^

Recent advancements in scRNA‐seq have enabled the identification of specific cell populations and their uniquely expressed genes, offering novel targets for diabetes treatment. In particular, Wang et al. utilised scRNA‐seq to discover a new type of endocrine progenitor cell in the mouse pancreas, highlighting its potential therapeutic roles in diabetes management.^74^ A single‐cell map of Type 1 diabetes (T1D) islets using scRNA‐seq was created and further reanalysed to identify T1D‐specific expression pattern of *CADM1* gene,^75^, ^76^ and validated with subsequent genetic and pharmacological investigations on the mechanisms of *Cadm1* in T1D progression. Moreover, Fukaishi et al. analysed pancreatic islets using a highly sensitive method, droplet‐assisted RNA targeting by single‐cell sequencing (DART‐seq), and identified a novel glucagon‐like peptide‐1 (GLP‐1)‐mediated pathway in humans that regulates α‐cell functions.^77^ Unlike other tissues, pancreas is one of the most technically challenging tissues for transcritpome analysis due to the high content of RNases. To capture rare cells from the islets of Langerhans, Muraro et al. developed an automated approach, integrating FACS, robotics and the CEL‐Seq2, and generated a single‐cell transcriptional map of the human pancreas, identifying heat‐stable antigen (HSA)/CD24 and transmembrane 4 L six family member 4 (TM4SF4) as marker genes for screening α and β cells.^78^ The reduced proliferative capacity of β cells during the postnatal period was investigated by creating a transcriptomic map of islet developmental trajectories and β cell state changes. A new algorithm was subsequently developed to construct a trajectory that describes the transcriptional changes occurring during islet formation in endocrine progenitors using this data.^79^, ^80^

## 4 CARDIOVASCULAR DISEASES

The prevalence of heart diseases has nearly doubled over the past few decades, increasing from 271 million cases in 1990 to 523 million in 2019. Traditional risk factors such as obesity, ageing, smoking, high cholesterol levels and hypertension significantly contribute to the rapid progression of coronary artery disease. Consequently, promoting cardiovascular well‐being remains a significant challenge for both clinicians and scientific investigators.^81^ Hypertension is a major risk factor for various cardiovascular diseases. A key event in hypertension is vascular remodelling, which increases vascular resistance. However, the exact molecular mechanisms driving this process are not yet fully understood.^82^ In a pioneering effort, using scRNA‐seq, Miao et al. created a cell map of the innermost layer intima of chronic thromboembolic pulmonary hypertension (CTEPH) patients’ pulmonary blood vessels.^83^ The analysis identified seventeen unique cell clusters, specified by a comprehensive panel of 10 518 marker genes. Eight distinct cell types, including stem cells expressing cysteine‐rich secretory protein LCCL domain, NK cells, lymphocytes, macrophages, endothelial cells and tumour cells, were identified from these clusters. Moreover, both the mesenteric artery and the aorta were subjected to scRNA‐seq to identify significant modifications in immune cells, smooth muscle cells, endothelial cells and mesenchymal cells. The study detected increased levels of certain cytokines within these cells, suggesting that immune cell proliferation induced by inflammatory cytokines may cause vascular changes during hypertension development.

Cardiomyopathy, a condition marked by heart muscle or electrical dysfunction, leads to heart failure and encompasses a variety of diseases where untreated degeneration of cardiac muscle cells and subsequent fibrosis are observed that impair diastolic heart function. Over the past few years, novel insights into the pathogenesis at cellular levels have been obtained with scRNA‐seq. Due to the scarcity of clinical samples, several studies have investigated the effect of genetic mutations on cardio lineage commitment and cardiomyopathy using induced pluripotent stem cells (iPSCs) models.^84^ Several studies have also investigated the somatic cell evolutions and cellular dysfunctions using clinical biopsies from failing hearts.^85^, ^86^, ^87^ For instance, by analysing nearly 600 000 nuclei from failing and non‐failing hearts with scRNA‐seq, Mark et al. have generated the first cellular landscape for single‐cell transcriptome for cardiomyopathy and identified a unique population of activated fibroblasts linked to the disease.^85^ An immune‐inflammatory atlas was created to study the progression of myocarditis to cardiomyopathy.^88^ Over 34 000 CD45^+^ cells from human hearts at premature, intermediate and final phases of clinical autoimmune myocarditis and animal models were analysed to identify 26 distinct cell types. Significantly, they discovered that groups of macrophages with elevated HIF1a‐regulated gene expression were intrinsically connected to tissue damage and inflammation.

Unlike cardiomyopathy, atherosclerosis (AS) is commonly recognised as an inflammatory disease. In fact, immune cells significantly impact atherosclerotic plaque development and progression. Upon ingesting oxidised low‐density lipoprotein, macrophages that line blood vessels turn into foam cells which can worsen the inflammatory response as they progress, rupturing to release damaging substances.^89^ As for other disorders, scRNA‐seq technologies have been utilised to establish the first comprehensive cellular map of healthy human heart arteries.^90^ Their cluster analysis identified ten primary cell types, with vascular smooth muscle cells (VSMCs) being the predominant type. Further gene expression profiling of VSMCs revealed four distinct subsets, out of which one subset was associated with enhanced migration and proliferation with higher expression of *FABP4*, as well as mitochondria genes *MT1A* and *MT1M*. This finding provides valuable insights into the mechanisms that may contribute to the development of atherosclerosis.^90^ Similarly, fourteen distinct cell types within atherosclerotic plaques were identified that displayed both smooth muscle cell and angiogenic markers, suggesting a transition from endothelial cells to mesenchymal tissue and the presence of angiogenic‐supporting cells.^91^ The myeloid cells harboured IL‐1‐producing pro‐inflammatory macrophages with inactivated inflammasomes, as well as TNF‐expressing macrophages and cells boasting foam cell and macrophage markers. In addition, another study investigated patrolling monocytes using scRNA‐seq technologies and highlighted the role of the *Lyn* gene in the origination and operation of the same.^92^

A significant contributor to acquired paediatric heart disease is Kawasaki disease (KD) which presents with systemic inflammation, vasculitis and an increased risk of coronary aneurysms.^93^ Studies using scRNA‐seq in KD patients showed that the proportion of naive CD8^+^ T cells, T helper cells and B cells was lower, while the proportion of mature T cells, B cells and NK cells was higher compared to healthy samples. The increased number of dendritic cells (DCs) in KD and the richer classification of subgroups suggest their essential roles in participating in and regulating the immune mechanism. In addition, the GO analysis conducted on B cells and T cells revealed that the lymphocyte activation was enriched in the regulatory pathways of the immune system. Zhou et al. further revealed 13 cell subgroups, including eight lymphocyte subgroups (two T lymphocyte subgroups expressing CD4^+^, three T lymphocyte subgroups expressing CD8^+^, two B lymphocyte subgroups and one natural killer (NK) cell subgroup) and five DC subgroups in KD.^93^ HLA‐DR, involved in blood cell differentiation, was found to be linked to immune regulation in both KD and some autoimmune diseases (AIDs), with susceptibility potentially tied to specific sites on the HLA gene (DM, DO, DP, DQ, DR).^94^ Altogether, the application of single‐cell transcriptome profiling methodologies has revolutionised our understanding of cardiovascular physiology and related disorders.

## 5 CANCER

The compositional complexity and clonal heterogeneity between cancers have made it challenging for scientists to study the disease as a whole. In cancers, the tumour microenvironment (TME) comprises both cancer and non‐cancerous cells, and their molecular mechanism of action modulates the development, progression and even chemoresistance to therapies. Several scRNA‐seq‐based landmark studies have been published in the past decade that unravel the heterogeneity, evolution, metastasis, therapy resistance and cellular interactions between TME and the immune system.^95^, ^96^, ^97^, ^98^ Generally, these studies are orchestrated to have a multifaceted approach that comprises three steps: Firstly, the selection of appropriate OMICS sequencing methods for investigating a particular tumour tissue. Secondly, looking at the tumour heterogeneity and unravelling immune subtypes and states that are specific to the TME, and finally, selecting a suitable therapy strategy that could throw insights towards selection, monitoring and response towards a particular treatment.^99^ This ensures deciphering the complexity of the disease, understanding therapy resistance and expediting the process of ‘bench to bedside’.

The prevalence of oncogenes and tumour suppressor genes within the tumour tissue could determine the status of tumour differentiation from primary to metastatic and thus provide insights on the required treatment strategies. Various other factors like an increase in the expression of intercellular adhesion molecules, extracellular matrix adhesion and tumour neovascularisation could be used as a sign of tumour metastasis.^100^ Dynamic changes due to the process of metastasis can also be observed in the cellular and transcriptional heterogeneity of the TME, as documented by several studies.^101^, ^102^, ^103^ Tumour, when metastasising, also gives rise to circulating tumour cells (CTCs) due to epithelial to mesenchymal transition.^104^ Testing for CTCs is thus used to monitor tumour dynamics, quantify the efficacy of treatment and identify the chance of recurrence in real time. Recently, a study used the overall and disease‐free survival of CTC‐positive and ‐negative lung cancer patients to identify a higher risk of recurrence in CTC‐positive patients.^104^ Studies on small cell lung cancer also showed that relapsed patients after platinum–etoposide treatment exhibited more unique CTC clusters, implying increased transcriptomic heterogeneity following treatment resistance.^105^ Furthermore, research on prostate cancer demonstrated heterogeneity in expression levels of oestrogen receptor genes. The activation of the non‐canonical Wnt signalling pathway in CTCs of patients undergoing anti‐androgen treatment is a sign of  resistance.^106^

During cancer progression, cancer stem cells (CSCs) are considered to be a major source of evolutionary selection that leads to recurrence, metastasis, heterogeneity and therapy resistance. Even though therapy‐induced secondary mutations and selection pressure could also cause clonal evolution and, in turn, enable resistance, it is observed that in most cases, a subset of these resistant cells is always a subpopulation of CSCs. Studies on lung cancer patients revealed the presence of a high‐plasticity cell state in the tumour that led to tumour progression.^107^ In gastric adenocarcinoma, tumour cells overexpressed stem cell transcription factor SOX9. This study suggested that targeting LIF and LIFR, regulated by SOX, could be a promising combinatorial therapy approach for gastric adenocarcinoma.^108^ Moreover, a pan‐cancer stemness gene signature was created using 34 scRNA‐seq data and corresponding CRISPR screening to establish the correlation between stemness and therapy resistance.^109^

However, there is an immense need to delineate lineage and temporal heterogeneity of the disease in order to develop better treatment options.  Using scRNA‐seq and cell‐lineage tracing analysis, researchers can reconstruct clonal lineage and understand the origin of specific cell populations and their biology. The genealogical status of gastric cancer, with 34 different cell lineage states, was identified using scRNA‐seq, with different states displaying distinct gene expression profiles. The data obtained from this study acted as a high‐resolution molecular resource for the genealogical states of several subtypes of gastric cancer.^110^ Also, another study on the transcriptomic profile of lung adenocarcinoma undergoing metastasis revealed the presence of a subtype of cancer cell that dominated the metastatic stage to be deviated from typical differential trajectory.^111^ Furthermore, various patient samples from prostate carcinogenesis, heterogeneous papillary renal cell carcinoma and Barrett's oesophagus leading to oesophageal adenocarcinoma have been used to identify the cell of origin for these cancers using SC‐assisted lineage tracing.^112^ This heterogeneity also applies to the immune cells in TME and is the basis for the development of cancer immunotherapy, one of the most effective methods which has witnessed significant advancement in recent years. Even though immune checkpoint inhibitor (ICI)‐based therapies are commonly used, the response towards the treatment depends on the tumour mutational burden.^113^ A study on 32 metastatic melanoma patients undergoing ICI therapy identified a subtype of CD8+ T cells as the predictor of favourable outcome.^114^ Multiple cytotoxic T cells undergoing clonal expansion were also found in ICI‐treated bladder cancer patients.^115^ Tumours with high mutational burden, like melanoma, lung, bladder and head and neck cancers, benefit from the treatment, while ones with low burden, like prostate, pancreatic and glioblastoma tend to be resistant as they exhibit reduced immune checkpoint expression.^116^, ^117^, ^118^ Using an integrative multi‐OMICS approach including scRNA‐seq, we recently discovered that the presence of extrachromosomal circular DNA (ecDNA) increases the genomic heterogeneity and correlates with an immunosuppressive phenotype and poor prognosis in high mutational burdening urothelial carcinoma.^119^ Biomarkers for chimeric antigen receptor (CAR) T cell therapy response have also been identified using scRNA‐seq technologies. Enrichment of memory and exhaustion phenotype in the CAR T cell of the infusion product led to better therapy response and disease progression, respectively.^120^

To date, according to clinicaltrials.org, several studies are ongoing that use single‐cell‐based analysis data for cancer drug discovery and development. Two of those studies apply scRNA‐seq to characterise the response of immune cells towards adjuvant multiple myeloma vaccines.^121^, ^122^ These studies depicted an increment in the myeloma‐reactive CD8+ T cells in the patients.^121^ The combination of BRAF inhibitors and immunotherapy was tested in a phase II clinical trial for colon cancer patients. In this study, they found that intrinsic immune modulatory factors like interferon I and II response, antigen presentation and chemokines required for T cell recruitment are elevated during the therapy, which in turn reduced tumour growth.^123^ Chemotherapy agents like Nivolumab and Ipilimumab were tested in combination with immune‐checkpoint inhibitors in operable lung cancer patients to observe more CD8+ T, CD4+ T, B and NK cells, and fewer regulatory T cells, exhibiting an anti‐tumour behaviour.^124^ The clonal heterogeneity of metastatic HER2‐positive oesophagogastric cancer was detected before and after treatment using scRNA‐seq technology to correlate higher heterogeneity to poorer outcomes.^125^ Multiple PI3K inhibitors that can initiate a tumour‐immune response are also in clinical trials for various cancers, with six approved and 35 ongoing.^126^ In conclusion, studying of cancer evolution, progression, TME and response to treatments using scRNA‐seq is one of the most broadly applied domains among all the clinical applications. Due to the great intertumour heterogeneity at genomic and cellular levels, the implementation of scRNA‐seq for precision oncology represents a promising solution for personalised medicine.

## 6 AUTOIMMUNE AND AUTO‐INFLAMMATORY DISEASE

Autoimmune diseases (AIDs) arise when the body's immune system erroneously targets and attacks its own tissues, organs or cells. This condition results from a breakdown in immune tolerance, where the immune system mistakenly recognises normal or altered self‐components—such as proteins, tissues or enzymes—as foreign.^127^ Environmental or genetic factors can trigger this dysregulated immune response, leading to tissue and organ damage. AIDs encompasses over 80 chronic conditions, impacting approximately 5% of the inhabitants in Western nations, mostly affecting women. Though some aspects of molecular targets and their interactions are known, the aetiology and pathogenesis of AIDs are not completely understood and necessitate comprehensive elucidation and investigation.^128^ One of the major AID is rheumatoid arthritis (RA), which occurs as a result of chronic inflammation in multiple synovial joints of the body. Long‐term studies have demonstrated that 80% of the affected patients are disabled after 20 years.^129^ Thus, identifying essential inflamed tissue cellular subsets and their activation states is pivotal for the discovery of novel therapeutic targets for RA. It is well known that subpopulations of synovial tissue exhibit diverse anatomical positions, transcriptome differences and distinct functions. Using customised low‐cost microfluidic instrumentation, Stephenson et al. analysed synovial tissues of five RA patients and identified 13 distinct cell types within the tissue, and interestingly the identification of fibroblast subtypes expressing high levels of RA drivers like cytokines (*CXCL12*), matrix metalloproteinases (*MMP2, MMP3*).^130^ Zhang et al, on the other hand, used 51 synovial tissue samples obtained from joint replacement and ultrasound‐guided biopsy of patients with RA and osteoarthritis (OA) to further identify 18 unique cell populations using scRNA‐seq, mass flow cytometry, RNA‐seq and flow cytometry.^131^ The novel cells observed within the tissues include THY1(CD90)^+^HLA‐DRA‐hi fibroblasts, IL1B^+^ pro‐inflammatory monocytes, ITGAX^+^ TBX21^+^ autoimmune‐associated B cells, PDCD1^+^ peripheral T helper cells and follicular helper T cells. IL‐6 expression in THY1^+^HLA‐DRA hi fibroblasts and IL‐1B production in pro‐inflammatory monocytes were found to be potential mediators of RA pathogenesis. Another study that used synovial tissue macrophages (STMs) identified phenotypic changes in patients with early/active, refractory/active and persistent remission of RA.^132^ Several others studies has also been conducted into the heterogeneity of the disease and describing the inflammatory nature of the cells within RA tissues.^131^, ^133^, ^134^, ^135^, ^136^, ^137^ To date, the most effective and common method for treating RA is by managing the chronic pain and inflammation using non‐steroidal anti‐inflammatory drug (NSAID). Ex vivo experiments showed that NSAIDs including naproxen prevent effective TNFα‐induced responses in HBEGF^+^ macrophages, suggesting the promotion of a classic pro‐inflammatory macrophage phenotype by NSAID therapy.^135^ Alivernini et al discovered that there are distinct subsets of synovial macrophages that regulate inflammation and remission in RA. Macrophage subpopulations displayed varied gene expression profiles among patients with early/active RA, treatment‐refractory/active RA or RA in sustained remission. MerTKpos macrophages exhibited gene expression signatures enriched in negative regulators of inflammation.^136^ In vitro, synovial fibroblasts were prompted by macrophages to produce inflammation‐resolving lipid mediators and initiate tissue repair. Low numbers of MerTKpos macrophages in RA patients in remission signal an elevated risk of disease recurrence. Altogether, utilising scRNA‐seq on relevant cell populations in RA has aided researchers in discovering precision treatments for the underlying mechanisms of persistent inflammation and joint destruction in disease.^138^

Like RA, systemic lupus erythematosus (SLE) is a chronic, remitting, relapsing systemic AID but can impact multiple organs including the skin, joints, lungs and kidneys. The immunological hallmark of SLE is the breakdown of tolerance against nucleic acids, marked by unforeseeable remissions and flares resulting in progressive organ damage. Lupus nephritis (LN) is the most common and severe manifestation, affecting approximately 50% of SLE patients, with 10% of these cases progressing to end‐stage renal disease. To date, there is only one new treatment that has been approved for treating SLE in more than 60 years.^139^ In 2017, a pioneering study employed scRNA‐seq in SLE,^140^ illustrating an up‐regulated IFN response in renal tubular cells of SLE patients and identifying a correlation between IFN‐response scores with chronicity index, IgG deposition and proteinuria. In agreement with this, another study using PBMCs from children with SLE, found elevated expression of interferon‐stimulated genes (ISGs) that can be used to distinguish SLE patients from healthy.^141^ A unique subset of ISG‐enriched and/or monogenic lupus‐related genes were identified in patients with higher disease activities.^141^ In addition, Zheng et al. analysed ten SLE patients using single‐cell 5′ RNA‐seq and sc‐TCR/BCR sequencing and identified an increase in TCR and BCR clonotypes, with a preference for certain V(D)J genes, in the SLE group. This study profiles the transcriptome and TCR/BCR immune repertoires in SLE patients, potentially opening new avenues for SLE diagnosis and treatment. The single‐cell immune repertoire and transcriptome obtained in this study through scRNA 5′‐end sequencing allow for more authentic and reliable joint analysis of the immune repertoire and transcriptome, yielding valuable insights into SLE disease mechanisms.^142^ Subsequently, Perez et al. developed mux‐seq, a cost‐effective and high‐throughput method for single‐cell sequencing of population cohorts and used it to study 1.2 million PBMCs from 162 SLE and 99 healthy control individuals.^143^ Moreover, sc‐ATAC seq studies in SLE patients have shown that their ancestral lineage could influence the immune cell composition and the subsequent disease status, which may explain differences in SLE susceptibility and severity among patients.^143^

Psoriasis is a lifelong, chronic immune‐mediated inflammatory skin disease with cutaneous and systemic manifestations. It is associated with morbidities such as psoriatic arthropathy, and psychological, cardiovascular, and hepatic diseases, leading to substantial negative effects on patient life quality.^144^ By analysing healthy inflamed skin samples with scRNA‐seq, Cheng et al. discovered an enrichment of CD1C^+^ CD301A^+^ myeloid DCs and CD3^+^ αβT cells in psoriatic epidermis while there was a reduction in CD207^+^ CD1A^+^ Langerhans cells suggesting that the proliferation and/or recruitment of epidermal DCs may further stimulate the activation of effector lymphoid and myeloid cells, contributing to the clinical features of psoriatic skin.^145^ Another study from Liu et al. identified two distinct, metabolically different, and CXCL13 expressing Tc17 subsets within CD8^+^ T cells from psoriatic skin via scRNA‐seq.^146^ In a support vector machine classifier comparing psoriasis and healthy transcriptomes, the CXCL13 showed comparable or superior accuracy to IL17A as an indicator of psoriasis severity. This indicates that an autoimmune‐induced CD8^+^ T‐cell response, driven by IL‐17, is central to psoriasis. Psoriatic Tc17 subset cells were identified and showed enhanced cytokine, cytolytic and metabolic transcriptional activity compared to melanoma‐infiltrating CD8^+^ T cells, distinct from the exhaustion profile. Bielecki et al. employed scRNA‐seq techniques to characterise skin innate lymphocytes (ILCs), revealing different states and proportions of these cell types across disease states. They identified a time‐dependent gene expression program with increased pro‐inflammatory signals, ILC3 and helper T cell‐17‐related genes.^147^ Psoriatic arthritis (PsA) is an inflammatory rheumatic disease, which commonly occurs in association with cutaneous psoriasis (PsO), with approximately one‐third of individuals with PsO developing PsA.^148^, ^149^ Single‐cell‐based studies on the synovial fluid of PsA patients observed an increment in the proportion of CD14^+^ CD16^+^ intermediate monocytes/macrophages, while there was a decrease in the proportion of CD14^+^ CD6^−^ classical monocytes/macrophages. These cells expressed PAR2 concurrently and their activation on monocytes/macrophages by tryptase‐6 led to the production of elevated levels of MCP‐1indicating the possibility of targeting the Typtase‐6‐PAR2 signalling pathway for therapy.^150^ Lately, Grivas et al. identified a distinctive ‘activity signature’ in PsA blood marked by TNF‐ and IFN‐induced inflammation, altered lipid metabolism and increased pro‐inflammatory non‐classical monocytes. The findings indicate an enriched ‘PsA‐specific gene set’ in extracellular matrix (ECM) metabolism, supported by an amplified interaction network between blood immune cells and skin mesenchymal cells. These results provide evidence of continuous TGFβ signalling and angiogenesis in treatment‐resistant PsA and suggest a gene expression signature linked to platelet activation and Hippo signalling as a possible marker of early treatment response.^148^

Systemic juvenile idiopathic arthritis (SJIA) is a common connective tissue disease in children, with chronic arthritis as its main feature, and can be accompanied by systemic multisystem damage.^151^ Utilising flow cytometry, bulk RNA‐seq, scRNA‐seq, DNA methylation analysis and Treg suppression assays, Julé et al. found that in synovial fluid, CD4^+^, CD8^+^ and γδ T cells expressed Th1‐related markers, unlike Th17 cells.^152^ Despite Th1 polarisation, Tregs retain regulated gene expression signatures by preserving Treg‐specific methylation patterns and functionality, these Th1‐like Tregs ensure the stability of their distinct Treg population in the joint.^94^ Another study on macrophages from sJIA‐MAS patient's bone marrow determined the existence of 11 distinct subpopulations, among which was an overrepresented IFN‐responsive cell population. A TRIM8‐marked population displayed a robust IFN‐γ‐induced response, characterised by substantial up‐regulation of immune and cytokine‐related pathways, coupled with an expanded transcription factor network. Particularly, pathways involved in intracellular granule movement were significantly up‐regulated in this macrophage population, including the MAS‐associated gene STXBP2 (syntaxin binding protein 2), verifying the correct identification of haemophagocytic macrophages. The robust IFN‐γ‐induced macrophage profile is consistent with the notion that IFN‐γ alone can interact directly with macrophages, inducing haemophagocytosis and leading to inflammatory desmoplastic anaemia. In cases like these, scRNA‐seq enables researchers to investigate the cell types with their functional states more thoroughly.^153^

Systemic sclerosis (SSc) manifests itself as a connective tissue disease with a pathogenic triad consisting of congenital and adaptive immune disorders, fibrosis affecting the skin and multiple internal organs (like heart, kidney and lung) and microvascular damage.^154^ Gaydosik et al. conducted droplet‐based scRNA‐seq on 3729 CD3^+^ lymphocytes, including 867 normal cells and 2862 cells from SSc skin samples.^155^ This study identified a unique cluster of recirculating CXCL13+ T cells in the SSc skin, expressing a T helper follicular‐like gene expression signature and associated with B‐cell responses within the inflamed skin. Confocal immunofluorescence microscopy confirmed the transcription results and showed the spatial arrangement of T cell subsets in progressive SSc skin samples. Additionally, Tabib et al. observed up‐regulation of PRSS23 and THBS1 in SSc fibroblasts. The identified upstream transcription factors, FOSL2, RUNX1, STAT1, FOXP1, IRF7, CREB3L1 and SMAD3, contribute to myofibroblast differentiation.^156^ Moreover, the relationship between APLNR and the angiogenesis disorder in SSc, as well as HSPG2's role in mediating the fibrotic response to vascular injury in SSc skin, has been theorised. Apostolidis et al. applied SmartSeq2 to revisit vascular endothelial cells from SSc skin, revealing novel markers APLNR and HSPG2. These findings suggest that APLNR may be related to the angiogenesis disorder in SScd, and HSPG2 may mediate the pro‐fibrotic response of SSc skin to vascular injury,^157^ providing promising targets for the development of treatments.

Ankylosing spondylitis (AS) presents with inflammatory lesions that tends to spread both upwards towards axial bones and downwards towards peripheral joints. In late stages, this condition can result in painful deformities and disabilities due to fibrous or bony fusion around the spine or hip joints.^158^ An early study of tissue samples from AS patients with lumbar spinal orthopaedics using scRNA‐seq discovered 26 cell subpopulations, including anti‐inflammatory M2 macrophages and stem cells undergoing high differentiation. The anti‐inflammatory M2 macrophages in AS enhance osteogenic differentiation of stem cell subsets by overexpressing VEGFA. Using bulk RNA‐seq, Li et al. detected abnormal up‐regulation of tenascin‐C (TNC) in ligaments and terminal tissues of AS patients. The TNC was found to promote new bone formation by enhancing chondrocyte differentiation and inhibiting ECM adhesion, thereby activating Hippo/YES‐related protein signals and increasing chondrogenic gene expression during endochondral osteogenesis. Moreover, scRNA‐seq and immunofluorescence staining revealed that TNC is predominantly secreted by fibroblast‐specific protein‐1 (FSP1)+ fibroblasts within the inflammatory microenvironment of the enthesis.^159^

Type 1 diabetes (T1DM) is another chronic AID typically affecting children and teenagers. Although not based on high‐throughput scRNA‐seq, Chiou et al. applied another highly attractive integrative genomics approach to acquire mechanistic insights into risk variants in T1DM. This study combined GWAS data from 520 580 T1DM patients and candidate cis‐regulatory elements (cCREs) in pancreas and PBMCs revealed by single‐nucleus assay for transposase‐accessible chromatin with sequencing (snATAC‐seq), and identified the overlap between risk variants and cCREs in T cells and other cell types.^160^ Using scRNA‐seq, Honardoost et al. identified 1784 genes exhibiting dysregulated gene expression in 13 immune cell types within PBMCs from stage III T1DM patients. The dysregulated genes are involved in WNT signalling, interferon signalling, T/NK cell migration, antigen presentation by B cells and monocyte activation, mirroring changes in T1DM pancreatic islets. In T1DM pancreatic islets, these genes that are differentially expressed were similarly altered. By constructing a T1DM metagene z‐score (TMZ) score utilising the scRNA‐seq data, they managed to differentiate cases and controls and assign patients into molecular subtypes. The score showed correlation with known prognostic markers in both immune markers and drug response in T1DM clinical trials. This TMZ score, which correlates with disease risk indicators and provides a basis for patient stratification, reveals significant heterogeneity in T1DM transcriptomic changes among patients.^161^

Inflammatory bowel disease (IBD), which comprises Crohn's disease (CD) and ulcerative colitis (UC), is a chronic autoimmune inflammatory condition of the gastrointestinal tract characterised by alternating periods of remission and flare‐ups. ScRNA‐seq of early human foetal intestinal samples, in vitro tissues, foetal organoids and epithelial cells from children's CD and healthy controls identified circulating BEX5+ epithelial precursor cells, revealed organoid maturation and detected disease‐related changes in epithelial components.^162^ This study also found that the reactivation of foetal transcription factors (TP53, MYC, HMGA1 and HMGA2) in epithelial cells of children with CD provides a wealth resource for studying intestinal development and children's CD.^162^ Mitsialis et al. conducted a study using mass spectrometry flow cytometry (CyTOF) technology and scRNA‐seq to analyse and compare immune cells in the colon mucosa and blood of patients with UC and CD and discovered distinct subpopulations of immune cells that were more abundant in the colon and blood of different patient groups, including those with active UC, active CD, inactive UC and inactive CD. Compared to control samples, the colonic mucosa of IBD patients showed increased levels of HLA‐DR^+^ CD38^+^ T cells, CXCR3^+^ plasmablasts, IL1B^+^ macrophages, and monocytes. In patients with UC, there was an increase in IL17A^+^ CD161^+^ effector memory T cells, IL‐17A^+^ regulatory T cells, HLA‐DR^+^ CD56^+^ granulocytes and a decrease in ILC3. On the other hand, CD patients exhibited elevated levels of IL1B ^+^ HLA‐DR ^+^ CD38^+^ T cells, IL1B^+^ TNF ^+^ IFNG ^+^ naive B cells, IL1B ^+^ DC and IL1B^+^ plasma cell‐like DC in their colonic mucosa. In the PBMC of patients with active CD, there was an increase in IL1B^+^ regulatory T cells, IL1B^+^ DC, IL1B^+^ plasmacytoid DC and IL1B^+^ monocytes, while ILC1 levels were decreased.^163^ To add to this, Li et al. conducted a study using scRNA‐seq on four inflamed tissues, four non‐inflamed tissues and colon tissues from four healthy individuals with UC. They identified 21 distinct cell groups and two previously unknown plasma cell groups. Their findings included: (1) A decrease in five cell groups within the inflamed tissues of UC patients, with an increase in three groups, specifically plasma cells. (2) The genes that showed significant differences in UC patients from China also showed similar patterns to those observed in patients from Europe and the United States, with an activation of the antigen presentation pathway and IL‐17 signalling pathway. (3) The primary immune marker for UC is Th17, but the most prevalent immune component in the affected tissues is immunoglobulin A, indicating a shift towards a local immune response. (4) Genes associated with UC are more prevalent in cells that give rise to epithelial tissue and immune cells.^164^ Devlin et al. performed scRNA‐seq on CD45+ haematopoietic cells from UC patients, both with and without ileal pouch‐anal anastomosis. They identified IL1B/LYZ+ myeloid cells and FOXP3/BATF+ T cells as markers of inflamed tissue. Additionally, they found that IL1B and S100A8/A9 myeloid cells interact with stromal cells and bacteria like *Bacteroides* and. In UC patients unresponsive to anti‐integrin therapy, the absence of a specific cell subset correlated with the presence of IL1B/LYZ+ myeloid cells.^165^

## 7 NEURODEGENERATIVE DISEASES

The chronic and progressive loss of cells from the central nervous system due to their structural and functional degeneration gives rise to neurodegenerative diseases like Alzheimer's disease (AD), Parkinson's disease (PD), Huntington's disease (HD), multiple sclerosis (MS) and amyotrophic lateral sclerosis (ALS).^166^ Even though many efforts have been made to understand the molecular mechanism of the pathogenesis, there are still no effective treatment nor biomarkers for early detection of the disease. Also, most of the neurodegenerative diseases are idiopathic, rather than genetic and are thus prone to share similar pathological and clinical characteristics.

Almost 95% of the PD patients, characterised by the loss of dopaminergic neurons (DaNs) and accumulation of fibrillar aggregates called Lewis bodies, are known to be idiopathic in nature.^167^ To gain further mechanistic insights into the disease, a previous study conducted scRNA‐seq of human mid‐brains with idiopathic PD and identified a specialised cluster of neuronal cells overexpressing CADPS2, a calcium‐dependent activator of secretion. Also, microglia, the primary immune cells in the brain, showed an increase in number as well as an increase in the expression of inflammatory genes.^168^ In substantia nigra, specific subpopulations of DaNs and oligodendrocytes were identified that exhibited disease‐specific gene profiles.^169^ Since most of the samples obtained for PD are from deceased patients, it is arduous to study the initiation of the disease. Hence, the development of iPSCs for in vitro studies has helped unshackle this issue a lot. Single‐cell technologies are now being used to compare the similarities of the cells differentiated from iPSCs to those found in humans and to study the onset of the disease as well as for disease therapy.^170^, ^171^, ^172^ HDAC4, a histone deacetylase, was found to act as a repressor of 60 genes that were found to be down‐regulated in PD. The use of pharmacologically modified HDAC4 is now used clinically to rescue PD‐related phenotypes and ER stress.^173^ Although the pathophysiologies of PD have been well studied, currently there is no therapeutic intervention that can modify disease progression.^174^ Recently, breakthroughs in stem cell‐based therapy of PD have been demonstrated in early clinical trials to be safe with disease benefiting outcomes.^175^, ^176^

AD is considered the most common neurological disorder, that causes dementia.^177^ The cellular and molecular interactions among the neurons, microglia and astrocytes are considered to be one of the pathophysiologies for the disease. By analysing 80 660 single‐nucleus transcriptomes from the prefrontal cortex of 48 individuals with varying degrees of AD pathology, Mathys et al. discovered that the disease‐associated changes appear early in the disease progression and exhibit high cell‐type specificity. This study also found that transcriptional dysregulations exhibited sex dependent, with females overrepresented in disease‐associated cell populations.^178^ Several studies have delineated AD progression in the past years.^179^, ^180^, ^181^, ^182^ A decrease in the number of B cells in the blood is found to be correlated to the dementia rating score in patients.^183^ On the contrary, in AD with tau pathology, the number of T cells increased leading to neuronal loss.^184^ Administration of T cell depleting antibodies decreased meninges and tau‐mediated neurodegeneration. Similarly disrupting APOE4 expression in neurons reduced tau pathology, neuronal degeneration and hyperexcitability.^185^

MS is a complex chronic central nervous system disease of unclear, multifactorial aetiology and lesion pathology, characterised by inflammation, gliosis, demyelination and neurodegeneration, lesions that affect the central nervous system's white and grey matter with focal inflammation.^186^, ^187^ Pappalardo et al, uncovered the immune milieu of the cerebrospinal fluid through scRNA‐seq and TCR sequencing in both healthy and neuroinflammatory individuals. T cells predominated in cerebrospinal fluid samples, while monocytes and DCs were also detected.^188^ Microglia heterogeneity has also been studied in MS lesional tissues from different developmental stages and brain areas. Among these, ten clusters emerged during development, two during demyelination and remyelination, and one during neurodegenerative disease. The transcription profile of microglia subtypes varies depending on the environment and developmental time, potentially revealing new drug targets.^189^ Moreover, Masuda T et al. utilised comprehensive methods like massively parallel scRNA‐seq, smFISH, IHC and computational modelling to characterise the subtypes of microglia in different brain regions during development and disease. Both the heterogeneity of microglia under steady state and the distinct molecular characteristics and cell dynamics of subtypes in various neurodegenerative diseases were identified, including that in MS.^190^ Interestingly, Clark et al. used RABID‐seq, which enables the concurrent examination of cell–body interactions and single‐cell transcriptomes, and studied MS patient samples to identify signalling pathways controlled by the axon guiding molecules Sema4D‐PlexinB1, Sema4D‐PlexinB2 and Ephrin‐B3/EphB3, which act as key mediators of microglia–astrocytic interactions that promote the pathogenesis of the CNS.^191^

## 8 INFECTIOUS DISEASES

The bacteria, viruses and fungi that make up the human microbiome work as a dynamic regulator of immune function. Depending on their innate traits as well as host environmental cues, many organisms can change between commensal, pathogenic or opportunistic behaviours. Since the relationship between microorganisms and the immune system can greatly affect disease susceptibility, development and consequences, it is imperative to comprehend this flexibility. For the advancement of focused therapy approaches and diagnostics for infectious diseases, a better understanding of this link is essential and this is approached through multiple methods of scRNA‐seq techniques.

The application of scRNA‐seq to bacterial systems has lagged due to a number of technical challenges specific to prokaryotic cells, such as the lack of poly‐A tails on bacterial mRNAs, low RNA content in individual cells and resistant cell wall structures, which necessitate the development of specialised techniques for efficient cell lysis, RNA extraction and mRNA enrichment in bacterial systems. Nevertheless, recent years have seen significant advancements in the development and improvement of bacterial scRNA‐seq methods, such as MATQ‐seq.^192^, ^193^ RamDA‐seq,^194^ PETRI‐seq,^195^ BacDrop,^196^ ProBac‐seq,^197^ M3‐seq,^198^ and smRandom‐seq.^199^, ^200^, ^201^, ^202^ These developments have created new opportunities for studying population dynamics, stress responses and bacterial physiology at unprecedented resolutions.^203^

Among these innovations, one particularly impactful advancement is BacDrop, developed by Ma et al., which effectively overcomes many of the technical limitations that have historically hindered bacterial single‐cell transcriptomics. By pairing universal rRNA depletion with a smart combinatorial barcoding system, BacDrop can process thousands to millions of cells from both gram‐negative and gram‐positive species simultaneously. When applied to clinical isolates of *Klebsiella pneumoniae*, the method revealed surprisingly high levels of gene expression diversity, even in populations that seemed uniformly driven in part by mobile genetic elements tied to antibiotic resistance. Under antibiotic treatment, BacDrop identified distinct transcriptional subgroups linked to different survival outcomes, including persistence, which would be missed by traditional bulk RNA‐seq. This approach offers a clearer window into how bacteria adapt and respond at the single‐cell level, with great potential for advancing our understanding of infection, resistance and the microbiome.^196^

Beyond profiling bacterial cells themselves, scRNA‐seq is increasingly being employed to map the presence and influence of microorganisms within human immune cells, an emerging frontier exemplified by the work of Yadav et al. Their study tackled the long‐standing challenge of detecting intracellular microbes particularly bacteria, viruses and fungi that reside within human immune cells in low abundance or are difficult to culture. Their research, however, examined PBMCs from participants who had recovered, SARS‐CoV‐2‐infected patients and healthy persons. Surprisingly, out of the 76 bacterial species they found, 16 had notable group‐to‐group variations in abundance. Some immune cell types, including memory B cells, regulatory T cells and naive T cells, were shown to be abundant in some microorganisms, including *Ehrlichia canis*, *Streptomyces clavuligerus* and *Buchnera aphidicola*. In individuals with COVID‐19, other bacteria, such as *Staphylococcus aureus* and *Leptospira interrogans*, were more prevalent, which raises concerns about their potential roles in immune regulation or disease severity. New understanding of the complex interactions between intracellular microorganisms and host immunity can be gained by identifying these cell‐specific microbial relationships. Understanding this relationship could help identify new treatment targets and guide creative infection control techniques.^204^

Single‐cell tools, such as Microbe‐seq, provide researchers with new ways to study how bacteria interact with human cells, especially in complex diseases such as cancers. In one study, Ghaddar et al. used Microbe‐seq to examine pancreatic tumour samples and identified 19 types of bacteria, including a high abundance of Campylobacter, a species known to cause inflammation. Interestingly, tumour cells showed more frequent contact with bacteria than other cell types, pointing to a possible role for microbes in shaping tumour behaviour and TME. The T cells in these tumours also had gene expression patterns similar to those seen during infection, which may help explain why the TME is so inflamed and why immunotherapy tends to be ineffective in these patients.^205^, ^206^ Another study by Avital et al. used ScdualNA‐Seq to examine both host cells and *Salmonella* inside them. They discovered two different groups of *Salmonella*, each with distinct gene‐expression profiles. Depending on the type of *Salmonella*, infected macrophages ended up in one of three states: partly activated by class I, fully activated by class I or fully activated by class II. These patterns followed a clear progression, showing that even subtle differences between bacteria can lead to different outcomes in host cells. Altogether, these findings highlight how single‐cell sequencing can reveal fine details of host–pathogen interactions that were previously hidden.^207^

A recent study used scRNA‐seq to examine PBMCs from people with active TB, latent TB infection (LTBI) and healthy controls.^208^ Major immune cell types, such as T cells, B cells and myeloid cells, were identified, which were grouped into 29 different subsets. This study identified consistent decrease of a single subset of NK cells from healthy individual samples to samples from LTBI and TB. Using flow cytometry, this depletion was verified, and the NK cell fraction found could be a helpful indicator to differentiate between latent and active TB, perhaps leading to better diagnosis and more focused treatment plans. In another study, Kazer et al. examined PBMCs from people sampled prior to and at various intervals following viral identification in order to investigate immune system alterations during acute HIV infection.^209^ Following the classification of common immune cell types and subsets, the team looked at how the phenotypes of these cells changed over the course of infection. They discovered that the expression of genes linked to NK cell mobility, DC activation, naïve CD4^+^ T cell maturation and monocyte antiviral activities were significantly correlated with viral load, increasing at the moment of virus identification and progressively decreasing as the infection advanced. Studies have also examined the impact of bacterial variability on immune responses in addition to viral infections. Avraham et al. investigated the response of macrophages to infection using scRNA‐seq and fluorescently tagged *Salmonella*.^210^ According to their investigation, the degree of PhoP/Q system activity in the bacteria was associated with two different patterns of type I interferon (IFN) activation. Macrophages that consumed Salmonella with increased PhoP/Q activity exhibited more robust type I IFN responses, most likely as a result of variations in the system‐regulated levels of surface lipopolysaccharide (LPS). Building on this, Saliba et al. investigated the effects of *Salmonella* replication rate variation on host cell behaviour. They found that macrophages infected with non‐replicating bacteria displayed pro‐inflammatory M1‐like signals, similar to uninfected bystander cells, using fluorescence reporters and transgenic bacteria. Those that carried fast‐replicating *Salmonella*, on the other hand, changed to an anti‐inflammatory, M2‐like phenotype, indicating that the dynamics of bacterial growth can rewire host cells to support bacterial survival.^211^

Latently HIV‐infected primary CD4^+^ T cells are transcriptionally diverse and may be divided into two major cell clusters, according to Golumbeanu et al.’s scRNA‐seq demonstration of host cell heterogeneity.^212^ Their unique transcriptional patterns are correlated with their propensity to restart HIV expression in response to stimulation. Specifically, 134 genes pertaining to RNA and protein metabolism, electron transport, RNA splicing and translational control were shown to be differentially expressed. CD4^+^ T cells recovered from HIV‐infected people further supported the results based on in vitro infected cells. Similarly, a number of potential Zika virus (ZIKV) entry receptors were investigated in the human developing cerebral cortex and developing retina using scRNA‐seq and immunohistochemistry, and AXL was found to exhibit notably high transcript and expression levels.^213^ Likewise scRNA‐seq can be employed for investigating disease pathophysiology and treatment by discovering possible target cells of novel pathogens. The human angiotensin‐converting enzyme 2 (ACE2) is bound by the spike protein of the virus SARS‐CoV‐2, which is the cause of the COVID‐19 pandemic.^214^, ^215^ Viral doorway is facilitated by this interaction in conjunction with the host protease type II transmembrane serine protease TMPRSS2.^216^, ^217^ Lung type II pneumocytes, ileal absorptive enterocytes and nasal goblet secretory cells have been identified to co‐express ACE2 and TMPRSS2 by examining the available human scRNA‐seq data, implying that they might be prospective targets of SARS‐CoV‐2.^218^

Extending these observations, the intratumour microbiome has become a new and quickly developing area of study due to the identification of microbes in a variety of cancer types, even in organs that are traditionally thought of as sterile, namely, in gastrointestinal tumours.^219^ Although the precise connection between them and cancer is still unknown, sequencing technology might be a useful remedy. Targeting a conserved region of the bacterial 16 S rRNA, Galeano et al. established invasion‐adhesion‐directed expression sequencing (INVADEseq), which made it possible to efficiently produce cDNA libraries with bacterial transcripts extracted from human cells associated with bacteria. The approach proves crucial for revealing the intricate relationships between microbiota and tumour tissues.^220^ Building upon these insights, the single‐cell analysis of host–microbiome interactions (SAHMI), a computational framework created to extract genuine microbial signals from host‐derived single‐cell RNA‐seq data, is another tool added to this toolset.^205^ SAHMI provides a system‐level perspective of microbe‐host interactions by profiling microbial species in relation to particular host cell types and connecting microbial presence to modifications in host gene expression. This capacity offers an expanded awareness of how microorganisms may affect host physiology and intercellular communication, especially at the single‐cell level.^205^ In the coming years, continued refinement and broader deployment of these technologies are poised to illuminate deeper layers of microbial complexity, fostering both conceptual breakthroughs and translational advancements.

## 9 EXPLOITING TRANSCRIPTOMICS DATA FOR DRUG DISCOVERY AND DEVELOPMENT: TILL NOW

The completion of the Human Genome Project has enabled unprecedented progress in the way researchers approached the studies towards drug discovery and development.^221^ Drug discovery, often regarded as an inefficient process, is burdened by high rates of attrition, extended timelines and rising consumption costs. The process by which a drug is introduced into the market is termed the drug development pipeline, which follows the strenuous steps of identification and validation of a disease target followed by the development of entities that will precisely target the molecule of interest. The introduction of OMICS platforms that improved the detection capability of transcripts at higher molecular levels has greatly improved the efficiency of this process.  These technologies reveal the transcriptional status of genes, their distinct regulatory patterns and molecular mechanisms that occur as a result of diseases and drug intervention. Consequently, high‐throughput sequencing technologies have become an integral part of medical science. The availability of numerous publicly accessible scRNA‐seq datasets further alleviates the decision‐making process while initiating research ideas, aiding in the identification of best practices and potential pitfalls (Table 1).

**TABLE 1:** A non‐comprehensive list of different databases and portals involved in scRNA‐seq approaches.

Name	Description	Accession links
**Databases**		
Source	Source is a web‐based database that brings together information from different range of resources and provides it in a useful manner for genome‐scale analysis	http://source‐search.princeton.edu/
NetAffx	NetAffx annotates probe sets on Affymetrix GeneChip microarray	http://www.affymetrix.com/analysis/index.affx/analysis/index.affxanalysis/index.affx
GenomeRNAi	The GenomeRNAi database contains phenotypes from published cell‐based RNA interference (RNAi) screens in *Drosophila* and *Homo sapiens*	http://www.genomernai.org/
ArrayExpress	ArrayExpress is a public database for HT functional genomic data.	http://www.ebi.ac.uk/arrayexpress/
GEO	The Gene Expression Omnibus (GEO) database is an international dataset that archives and distributes high‐throughput gene expression and other functional genomics datasets.	http://www.ncbi.nlm.nih.gov/geo/
SC2disease	Manually curated database of single‐cell transcriptome for human diseases	http://easybioai.com/sc2disease/
Autoimmune Diseases Explorer (ADEx)	Transcriptomics and methylation datasets for common auto immune diseases	https://adex.genyo.es
Genotype‐Tissue Expression project (GTEx)	Provides homogeneously processed datasets for tumour samples	http://www.gtexportal.org/home/)
PlaqView	Open‐source web portal for atherosclerosis single‐cell datasets	https://www.plaqview.com
PlaqView 2.0	Provides cardiovascular single‐cell datasets	https://www.plaqview.com
Drug Gene Interaction Database (DGIdb v4)	Explores current drug databases in the context of relevant single‐cell datasets	https://www.dgidb.org/
DISCO	A database of Deeply Integrated human Single‐Cell Omics data	https://www.immunesinglecell.org/
cBioportal	Cancer database	https://www.cbioportal.org/
Heart Cell Atlas	Provides heart cell states defined by sc/snRNA‐seq data and spatial omics	https://www.heartcellatlas.org/
Express Heart	Single‐cell RNA‐sequencing datasets for non‐cardiomyocytes	http://shiny.bios.unc.edu/expressheart/
scMoresDB	Comprehensive database for respiratory disease	https://ngdc.cncb.ac.cn/databasecommons/database/id/9060
PANC‐DB	Data portal of The Human Pancreas Analysis Program	https://hpap.pmacs.upenn.edu/
ssREAD	Single‐cell and spatial RNA‐seq database for Alzheimer's disease	https://bmblx.bmi.osumc.edu/ssread/
Allain brain map	Human and mouse brain datasets	https://portal.brain‐map.org/atlases‐and‐data/rnaseq
SCDEVDB	Single‐Cell Developmental Data Base	https://scdevdb.deepomics.org/
scREAD	Human/mouse (Alzheimer's) scRNA datasets	https://bmbls.bmi.osumc.edu/scread/
SCAD‐Brain	Public database of single‐cell RNA‐seq data in human and mouse brains with Alzheimer's disease	https://www.bioinform.cn/SCAD/
NTCdb	Single‐cell transcriptome database of human inflammatory‐associated diseases	www.ntcdb.org.cn.
Interactive SummarisedExperiment Explorer (iSEE)	Enables users to host their SummarisedExperiment data to visualise their single cell data	https://rdrr.io/bioc/iSEE/
human Antigen Receptor database (huARdb)	A large‐scale human single‐cell immune profiling database	https://huarc.net/database
CancerSEA	Utilises single‐cell data from cancer datasets to decode the functional states of cancer cells	http://biocc.hrbmu.edu.cn/CancerSEA/home.jsp
Pig Cell Atlas	A single cell transcriptome atlas of pig cells	https://dreamapp.biomed.au.dk/pigatlas/
Data portals		
International Cancer Genome Consortium (ICGC)	Global effort covering various cancer types	https://dcc.icgc.org/
Metabric (Molecular Taxonomy of Breast Cancer International Consortium)	Breast cancer data sets	https://www.mercuriolab.umassmed.edu/metabric
Pan‐Cancer Analysis of Whole Genomes (PCAWG)	Multiple, global initiatives analysing whole cancer genomes	https://dcc.icgc.org/pcawg
Genomics of Drug Sensitivity in Cancer (GDSC)	Various cancer cell lines with drug response information	https://www.cancerrxgene.org/
Cancer Cell Line Encyclopedia (CCLE)	Information on various cancer cell lines	https://portals.broadinstitute.org/cc
Neuroblastoma Research Consortium (NRC	Neuroblastoma	https://nrc.rockefeller.edu/
Therapeutically Applicable Research to Generate Effective Treatments (TARGET)	Paediatric cancers (neuroblastoma, acute lymphoblastic leukaemia)	https://ocg.cancer.gov/programs/target
Asian Cancer Research Group (ACRG)	Gastric cancer	http://gigadb.org/dataset/100034
Pan‐Cancer Analysis of Whole Genomes (PCAWG)	Multiple, global initiatives analysing whole cancer genomes	https://dcc.icgc.org/pcawg
The Cancer Genome Atlas (TCGA)	Breast, lung, colon, glioblastoma	https://www.cancer.gov/ccg/research/genome‐sequencing/tcga
Genomic Data Commons (GDC)	Multiple aligns and harmonises data from TCGA and other projects	https://gdc.cancer.gov/
Asian Cancer Research Group (ACRG)	Gastric cancer	http://gigadb.org/dataset/100034
Interactive Analysis and Atlas for Autoimmune disease (IAAA)	Data's of single cell and bulk RNA seq of six autoimmune diseases	http://galaxy.ustc.edu.cn/IAAA
CellHub	Open‐access single‐cell data repository	https://cellhub.nygen.io/
HubMAP	Human BioMolecular Atlas Program Data Portal	https://portal.hubmapconsortium.org/
Single cell portal	Consortium of single cell data of most of the species	https://singlecell.broadinstitute.org/single_cell?order=recent
CZ CellxGene Discover	Collection of curated interportable single‐cell transcriptomic data	cellxgene.cziscience.com
Tumour Immune SC Hub (TISCH)	Repository of uniformly processed human and murine scRNA‐seq data covering several cancer types	http://tisch.comp‐genomics.org
ICARUS v3	Utilises the geometric cell sketching method for data analysis	https://launch.icarus‐scrnaseq.cloud.edu.au
SCircle	An interactive visual exploration tool for scRNA‐seq data, including bacterial scRNA‐seq	https://www.rna‐seqblog.com/scircle‐an‐interactive‐visual‐exploration‐tool‐for‐single‐cell‐rna‐seq‐data/
BacSC	Workflow for bacterial single‐cell RNA sequencing data analysis	https://johannesostner.github.io/publication/2024‐06‐27‐BacSC
Broad Institute Single Cell Portal	Provides access to a wide range of publicly available single‐cell datasets, including those generated from bacterial scRNA‐seq experiments	https://bigomics.ch/blog/ultimate‐guide‐to‐public‐rnaseq‐and‐sc‐rna‐seq‐databases/ https://singlecell.broadinstitute.org/single_cell
Vulture	Data portal for bacterial scrna seq analysis	https://academic.oup.com/gigascience/article/doi/10.1093/gigascience/giad117/7513487

Several high‐throughput technologies like transcriptomics, proteomics, metabolomics, glycosilomics, interactomics, phenotype screening, in silico data mining, genetic engineering and somatic mutagenesis are being implemented in human pathology studies to identify novel targets. Integrating information using these OMICS studies can identify different cell types and their effector genes that have an influential role in disease, providing mechanistic insight into potential therapeutic approaches. Due to its ability to explore the complex microenvironment of the tumour tissue, these integrative studies have a significant application in target identification in the field of oncology. Potential tumour antigens have been mapped recently as an effort to develop better‐targeting strategies against tumour cells, especially in haematological cancers that use monoclonal antibodies to deplete specific cells.^222^ Besides cancers, NKD2 was identified as a candidate therapeutic agent in human chronic kidney disease. Integration of GWAS data with sc‐RNA seq data was used to identify novel molecular signalling pathways in various diseases like major depressive disorder, ulcerative colitis and MS.^223^ Details on differentially expressed genes in particular diseases, when analysed in combination with known protein‐protein interaction networks, gave rise to GuiltyTargets, a computational framework that can predict new targets. Several artificial intelligence‐based models have also been created to predict such targets in various diseases like cancer, arthritis, and so forth.^224^, ^225^ However, these prediction models produce a myriad of data that will need to be credentialed before their validation in in vitro and in vivo models, as target validation is a time‐consuming and an expensive process.

Single‐cell CRISPR (scCRISPR) perturbation screening approach allows functional studies on multiple cell types, by enabling several genetic perturbations to be profiled simultaneously, making it a reliable tool for target credentialing. This, when combined with functional assays and Perturb‐seq, has enabled the identification of several targets for immunotherapy in cancer.^226^ The scCRISPR, in recent years, has also been incorporated with the possibility of CRISPR activation (CRISPRa) and CRISPR interference (CRISPRi) screening, which along with the possibility of scRNA‐seq profiling has widened the horizons of target validation. Functional genomics studies could be further used to credential the targets obtained. This and other pharmacological studies done in vitro and in vivo could enable the delineation of the molecular mode of action of these targets at higher resolution if combined with OMICS studies. However, molecular target‐based screening is advantageous over phenotypic screening as knowledge about a molecular target and its related screening assay enables easier optimisation of required concentrations and enables identification of toxicity levels and, subsequently, biomarker development.

Target credentials and validations are the most critical steps on the path to clinical translation. Several in vitro models, like cell lines, iPSCs and organoids, exist and are highly appreciated for their easily manipulatable and cost‐effective nature. For example, the Cancer Cell Line Encyclopedia, a siRNA seq data‐based profile of different cancer cell line models, is used to test therapeutic strategies disregarding cellular heterogeniety.^227^ Meanwhile, human organoids, among in vitro testing modalities, are a prospective model to replicate the human body. Studies on liver injury and pancreatic duct adenocarcinoma have displayed this promising nature of organoids.^228^, ^229^ However, the need for more complex biological systems to study cellular and ligand‐receptor interactions better diminishes the clinical translatability of in vitro‐based studies. Thus, scientists prefer in vivo models like syngeneic mice, patient‐derived xenograft (PDX) in immunodeficient mice and genetically engineered mouse models (GEMMs), which can replicate human disease expression profiles when used to create single‐cell transcriptomics data. This, too, is only possible in cases where the murine profile is comparable to the human expression profiles. Further, ethical concerns and protection of animal welfares have now called for the replacement, reduction and refinement of animal models in drug discovery. Thus, the best option is to use better human and disease‐mimicking systems, such as organoids or organotypic cultures. With the completion of single‐cell transcriptome atlases for various tissues and conditions, the conception and realisation of ‘virtual cells’ artificial models have now developed. In fact, several machine learning‐based approaches have leveraged the single‐cell omics data available from tumour biopsies to predict molecular targets related to drug sensitivity such as scDRUG, beyondCell, scDEAL, NCI‐DOE and DREEP.^230^, ^231^, ^232^, ^233^, ^234^, ^235^, ^236^

Transcriptomics has also revolutionised the patient stratification process. Identification of drug response‐based markers from clinical samples has steadfast the process of validating drug response. For instance, the identification of a cellular module called GIMATS using scRNA‐seq in inflamed tissues from patients with CD who underwent anti‐tumour necrosis factor (TNF) therapy enabled the measurement of remission score.^237^ Expression profiling of UC patients in comparison with healthy individuals identified immune and stromal cells that were responsible for resistance to anti‐TNF therapy.^238^ ScRNA‐seq has also been applied in measuring drug response by detecting the levels of minimal residual disease (MRD). The accuracy of MRD detection using the technology is being used to identify relapse‐causing multiple subclones in AML.^239^ Watermelon, a high complexity barcoded lentiviral library was created that could trace the clonal lineage of these persister cells during the course of treatment.^240^ It identified rare groups of cells that can proliferate under the influence of the drug with the help of antioxidant genes and metabolic shifts, which is now a major target against cancer recurrence. Consequently, the PERSIST‐SEQ consortium (https://persist‐seq.org/) and HTAN, were created which improved our understanding of therapeutic resistance during cancer.^241^ Similarly, expression profiling from multiple pre‐ and post‐treatment patient samples has been utilised to comprehend the mechanism of drug resistance at cellular resolution, especially in oncology.^96^, ^242^, ^243^, ^244^, ^245^, ^246^

## 10 EXPLOITING TRANSCRIPTOMICS DATA FOR DRUG DISCOVERY AND DEVELOPMENT: CHALLENGES

Despite single‐cell‐based OMICS analysis having provided an impressive range of discoveries in understanding the molecular mechanism and drug operatability during diseases, translation of the obtained insights into the clinical settings is still in its infancy. The majority of this delay in application owes to the fact that the scRNA‐seq techniques face several challenges between the path from ‘bench to bedside’. Regardless of the assays used or data modalities obtained thereafter, the generation and analysis of single‐cell data presents with numerous obstacles that must be addressed by researchers in both the planning and execution phase of their studies, as well as the high demand of data analysis and interpretation.

### 10.1 Technical limitations

Due to the limited availability of nucleic acid content within an individual cell, scRNA‐seq encounters several challenges in the procurement and amplification of RNA. These challenges include biases introduced during PCR amplification, dropout events and allelic imbalance, all of which result from the scarcity of initial RNA material. These experimental artefacts are currently tackled using the available bioinformatic QC tools. However, these methods could introduce further mathematical artefacts and affect the reproducibility of marker gene detection and subsequent analysis.^247^ Also, the high cost of the method creates a dilemma in deciding whether to acquire more coverage using fewer cells or shallower coverage using a higher number of cells, providing higher transcriptional resolution and unravelling diversity of cellular population, respectively. Often, as a result of interest in discovering rarer cell populations, researchers prefer to sequence a greater number of cells at lower coverage but in clinical settings, since patient samples are precious, cells are FACS sorted and sequenced at higher depth to detect previously unknown transcriptional programs.^248^ To tackle this, the Satija Lab has introduced an online calculator (https://satijalab.org/howmanycells/), provided researchers have prior knowledge about the diversity and relative composition of cells within the tissue under investigation, can predict the number of cells that need to be sequenced. Another major issue faced while dealing with many samples is the batch effects that appear during data generation and integration. Higher batch effect samples tend to have cells obtained from similar settings to exhibit higher transcriptional similarity. This is also observed while integrating several publicly available datasets. Several solutions have already been proposed that can tackle the issues during batch effect correction and dataset integration, however it needs to be noted that batch effect correction can lead to the disappearance of biological variations in the cells that might occur as a result of age or disease.^249^

### 10.2 Data variability and availability

To date, most of the published single‐cell transcriptomics datasets are publicly available. However, the absence of a standardised protocol in data generation creates considerable variability in the data format and layouts available online. This issue is further compounded by non‐uniform processing of the count matrix and the variability in cellular annotation methods employed by different laboratories, stemming from the lack of a well‐defined and standardised cellular nomenclature. The fact that the process of data generation and analysis is expensive and time consuming, warranties the need to avoid duplicate data generation. The generation of public repositories that are uniformly processed and integrated like Human Cell Atlas, and the Human Cell Landscape has helped avoid this issue to a great extent.^250^, ^251^ Such atlases also provide a starting point for researchers to discover datasets and jump the hurdle of manually searching PubMed and omics repositories.

## 11 EXPLOITING TRANSCRIPTOMICS DATA FOR DRUG DISCOVERY AND DEVELOPMENT: THE WAY AHEAD

### 11.1 Third generation sequencing (TGS)

scRNA‐seq has enabled depiction of gene expression levels within individual cells with an unmatched level of detail, offering important information on the status of each cells. Yet, existing scRNA‐seq primarily focuses on identifying read counts at either the 3′ or 5′ termini of polyadenylated transcripts, lacking adequate coverage of mRNA splicing. TGS, or long‐read sequencing, is an innovative molecular sequencing method that addresses this issue by detecting the entire length of cDNA and RNA in real‐time. In contrast to other NGS technologies, TGS can detect intricate DNA structural variations, whole transcript selective splicing events, and cell‐type‐specific mRNA isoform expression. Some TGS methods, like single‐molecule real‐time (SMRT) sequencing and nanopore sequencing, have been applied in scRNA‐seq studies to gain deeper understanding of selective splicing regulation, transcriptome complexity and isoform diversity in tumour cells at a single‐cell level. For instance, The TGS technology RAGE‐Seq was used in breast cancer to reveal complete antigen‐receptor sequences accurately for studying the clonal evolution of tumour‐associated B cells. Simultaneous genomic and transcriptomic detection on HCC samples identified structural variants and extrachromosomal DNA using the single‐cell parallel genome and transcriptome sequencing (scGTP‐seq) platform. Nanoranger, a new single‐cell TGS technology, improved the resolution of leukaemia and immune cell phenotypes in acute myeloid leukaemia patients. Integration of CRISPR and single‐cell TGS allows for functional gene characterisation.^252^ But, this too has its own challenges, like lower sequencing depth leading to expression level quantification and data sparsity, but tools like scNanoGPS have been developed that can optimise accuracy and reduce dependence on short‐read sequencing results.

### 11.2 Temporal modelling using transcriptomics data

The fact that biological systems and processes are dynamic questions the sufficiency of transcriptomics in understanding disease progress. As scRNA seq are often done at specific time points, the use of the data raises several challenges in conducting time‐series‐based studies, especially because, in scRNA‐seq studies, individual cells collected at a unique time point can represent a relatively wide range of cell states. This subsequently gives rise to several computational issues during analysis like the representation of cells collected over a time‐period, network inference and irregularity in recognising the timing of specific events.^253^ To overcome this, researchers use trajectory inference or RNA velocity^254^ to detect the timing and ordering of the events called pseudo‐time ordering. However, the computational capacity of existing algorithms used to detect these are limited and thus may lead to faulty reconstruction of the temporal ordering. Therefore, it is necessary to modify the experimental setup to obtain time‐series data as complementary to the computational tools. A conventional way to do this would be to collect tissue samples from patients at discrete intervals. Studies have used tissues from breast cancer patients before and during anti‐PD1 treatment to identify immunophenotypes and several genes corresponding to these phenotypes that could help predict treatment responses.^255^

One of the major ways to modify the experimental setup to study temporal ordering is to infer the relative age of the mRNA transcript being sequenced. Recently, metabolic labelling of nascent RNA with 4‐thiouridine or 6‐thioguanine like scSLAM‐seq, NASC‐seq and scNT‐seq has been enabling trajectory inference at higher resolution.^256^, ^257^, ^258^ This when combined with the knowledge on old transcripts enables identification of dynamic change in gene expression during the experimental time window. Chrono labelling is another strategy that uses cell type‐specific reporters to generate real‐time anchors, thereby improving the accuracy of trajectory detection.^259^ GESTALT (genome editing of synthetic target arrays for lineage tracing) is a modification of labelling strategy that uses CRISPR‐Cas9 to induce genetic mutations in cells, which could be used for lineage tracing during diseases.^260^ Live‐seq is another method that can directly map cellular trajectory by sequentially sampling cytoplasmic biopsy of the same cell without inducing major cellular perturbations.^261^ However, most of these techniques have been applied on only isolated cells or cell lines, which is disadvantageous as cellular phenotypes change when cultured ex vivo. Thus, there is an immense need to establish trajectory recording techniques that could be done in vivo.^262^

### 11.3 Expanding the dimensions using spatial transcriptomics

One of the major disadvantages of using scRNA‐seq is that it requires the cells or nuclei to be isolated from the tissue and lysed to retrieve RNA. This leads to the loss of potential spatial information that is imminent in understanding the cellular interactions within the tissue of interest. Thus single cell spatial transcriptomics technology has emerged at the frontier of molecular biology as an integrator of spatial information and the transcriptome.^263^ Several approaches were considered to improve the quality of this technique over the last decade. Platforms like MERFISH and seqFISH are of a targeted nature, and can measure a subset of the transcriptome with subcellular resolution.^264^, ^265^ Untargeted scST platforms like Visium ST and GeoMX DSP can detect the whole transcriptome but have lower resolution power. However, recently introduced Visium HD and Stereo‐seq have reached subcellular resolution.^266^, ^267^ Several deconvolution techniques exist that can be applied to understand these large amounts of spatial transcriptome results extensively.

Spatial transcriptomic technology has already begun to make a significant impact in the field of developmental biology and oncology. The analysis of gene expression patterns within the disease tissue and their cellular interactions while leveraging established biological databases can tune the drug screening processes to precision. For example, spatial transcriptomic analysis of human cutaneous melanoma identified potential target genes associated with cancer‐associated fibroblasts cells. These data were further used to identify drugs like mifepristone and dexamethasone using molecular docking.^268^ This demonstrates that these advanced transcriptomic techniques can be applied to discover new pathways that influences the diseases and can help expand the therapeutic scope of existing drugs and enable drug repurposing, thereby diversifying their application value. Furthermore, the combined application of classical transcriptomics and spatial RNA sequencing decoded the increment in the interactions between tumour and immune cells after NP137 treatment, establishing the inhibitory effect on Netrin‐1 and subsequent reduction of EMT.^269^ Garrido‐Trigo et al. deployed CosMx Spatial Molecular Imaging to IBD patient samples and localised each macrophage and neutrophil subset identified by scRNA‐seq in human tissue and revealed additional macrophage diversity based on their tissue location. ScRNA‐seq in conjunction with spatial transcriptomic analysis indicated a robust communication network between macrophages and inflammatory fibroblasts during IBD and established that myeloid and stromal compartments are important cellular subsets in explaining the patient‐to‐patient diversity during the disease.^270^ Spatial transcriptomics can also be applied to precisely identify and delineate the therapeutic possibilities suitable for individual patients and also validate their drug response. In a recent study on prostate cancer, scientists revealed thirty six ageing‐related microenvironment regulatory factors and three regulatory patterns that could be applied to develop personalised therapies.^271^ Other than cancer, studies on diabetic patients’ kidney tissues revealed previously unknown gene expression characteristics related to neovascularisation in the glomeruli, leading to novel personalised diagnostic prospects using renal biopsies.^79^ A spatial transcriptional map of human adipose tissue was also created that revealed the existence of a subpopulation of adipocytes associated with insulin sensitivity, indicating the potential in targeting this cell subtypes to modulate insulin resistance in adipose tissue, offering potential new avenues for diabetes treatment.^80^, ^272^ Integrating spatial transcriptomics with scRNA‐seq and temporal transcriptomics data has improved the studies using organogenesis as well. A precise reconstruction of the differentiation of retinal neuron and their localisation during organoid development was revealed using this integrated study, inferring the regulations of gene networks underlying these organoid developments.^273^, ^274^ Even for physiological conditions in humans, we recently demonstrated the value of spatial transcriptomics in identifying the regenerative niche of ageing human skeletons.^275^ Thus, in general, scST is being widely accepted and applied in the medical world and is being modified rigorously to attain perfection. The immense possibilities of using this technique have been discussed in detail elsewhere.^276^

### 11.4 Compiling multiple OMICS data for holistic view

Multi‐omics is a technique that can combine data obtained at different biomolecular level to provide a holistic view of the cellular and organ function. It is a fact that the complexity of human biology is beyond the scope of investigating it through a single‐omic technique. Multi‐omics, a technique that combines the data from genomics, proteomics, metabolomics, transcriptomics, perturbational, temporal and epigenomics is thus being popular in the field of biomedical research. Several studies have been put forward that successfully integrated several omics data to create meaningful conclusions to scientific research problems. For example, integration of genomics, proteomic, epigenomic and transcriptomics data from AD patients helped identify protein–protein interaction and gene expression patterns that are specific to the diseases.^277^ Multi‐omics data obtained from immune cells that infiltrate TME have been used to develop a deep‐learning platform that could predict drug response and patient survival during breast cancer.^278^ Frequent mutations in TRAF7 and KLF4 were also identified in meningioma using multi‐omics data obtained from patient samples.^279^ The single‐cell CRISPR screening methods can combine pooled CRISPR screening with single‐cell multi‐omics or scRNA‐seq. One such technology is Perturb‐seq.^280^, ^281^, ^282^, ^283^ which can look into the impact of individual perturbations on gene expression, their interactions, or their dependence on cell states, and can be further decoded using a number of computational frameworks (MIMOSCA, scMAGeCK, MUSIC, Mixscape),^280^, ^283^, ^284^, ^285^ as well as a screening platform. The most sensitive cell types to CRISPR‐mediated perturbations can then be prioritised at the single‐cell level.

Single‐cell DNA sequencing methods have been primarily employed to track cells with treatment‐resistant mutations and to infer the cell lineage of malignancies. Several computational methods^286^, ^287^ have been developed for the identification of single‐nucleotide variations (SNVs), short insertions and deletions (indels) and copy number variation (CNV) in order to get over technical restrictions such as non‐uniform coverage depth in scRNA‐seq. The CNV detection techniques for other technologies, such as whole‐genome sequencing (WGS) or whole‐exome sequencing (WES), array‐CGH, single‐nucleotide polymorphism (SNP) arrays and others, were also extended and used along with scDNA‐seq data.^288^ Consequently, utilising scRNA‐seq data, computational techniques like CopyKat and InferCNV have been developed to evaluate copy number and intratumoural heterogeneity.^289^, ^290^ In order to more clearly distinguish between host and cancer cells, the same techniques are also utilised to infer aneuploidy in cells using scRNA‐seq cancer data sets. Furthermore, functional mutations that control the expression of particular genes specific to cell types can be found using scRNA‐seq‐based point mutation detection techniques,^291^, ^292^ which enable the connection of genotype to phenotype. The best methods for mapping quantitative trait loci (sc‐eQTLs) with single‐cell expression have also been evaluated.

Single‐cell T cell receptor and B cell receptor sequencing technologies (scTCR‐seq and scBCR‐seq) enable detailed investigation of the dynamics of T cell and B cell clones in tissues or peripheral blood by determining clonotypes at a single‐cell level. Clonotypes refer to cells from the adaptive immune system that originate from a common ancestor and thus share the same TCR or BCR. TCR and BCR repertoire reconstruction and clonality inference can also be achieved based on scRNA‐seq using computational methods.^293^ Tools such as scRepertoire^294^ and CellaRepertorium facilitate the examination of clonotype dynamics. By coupling scTCR‐seq or scBCR‐seq with scRNA‐seq, researchers can uncover the relationship between clonotype and phenotype (or transcriptional states) in T or B cell populations.^295^ The detailed characterisation of T and B cells provided by single‐cell technologies has advanced the understanding of various diseases, such as cancer and MS, and has contributed to the improvement of engineered T cell therapies, including CAR T cells.

Various single‐cell sequencing technologies capture epigenetic characteristics at near‐nucleotide resolution. Single‐cell assay for transposase‐accessible chromatin sequencing (scATAC‐seq)^296^ reveals open chromatin structure, while scCUT&Tag and scChIP‐seq detect chromatin histone modifications, and scBS‐seq identifies DNA methylation patterns.^297^, ^298^ Understanding the promoters and enhancers activated in specific cell types or states helps identify the tissues, cell types and biological conditions where a target is abundantly expressed, as well as the transcriptional programs leading to its expression. Additionally, these techniques supports the identification of causal non‐coding variants associated with diseases discovered by genome‐wide association studies (GWAS), mapping them to specific cell types. Emerging single‐cell proteomics (sc‐proteomics) methods decode proteome variation across individual cells.^299^ These methods typically focus on either the absolute quantification of a small number of proteins or highly multiplexed protein measurements. A recently proposed method counts single proteins in single cells using nanopore single‐molecule peptide reads, which are sensitive to single‐amino acid substitutions within individual peptides. This advancement opens the possibility of developing single‐molecule protein fingerprinting in the future.^300^

**FIGURE 4:**
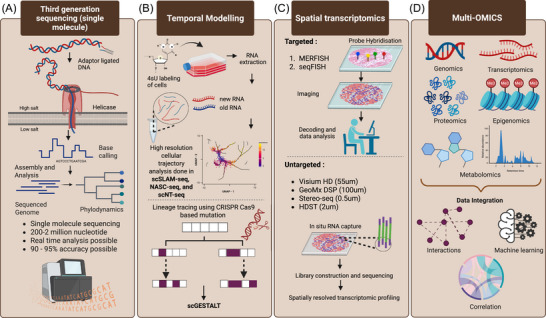
Schematic representation of how the scRNA sequencing techniques could be integrated with the existing experimental and computational tools to broaden our diseases understanding and pave way for better drug discovery using clinical data. (A) Third generation sequencing is distinguished from the previous generation by its ability to inspect single molecules individually, devoid of any wash steps. Nanopore technologies, as depicted in the figure can use the differential salt gradient across the membrane to measure the translocation of nucleotides across the pore, enabling real‐time data acquisition with approximately 95% accuracy. (B) Temporal modelling using metabolic labelling of cells at discrete time points enable temporal ordering of cells during disease progression and treatment. Several techniques like scSLAM‐seq, NASC‐seq and scNT‐seq uses this. Lineage tracing using CRISPR‐cas9‐based techniques like GESTALT is also popular, where the genetic mutations are introduced at different time points, that could also be used for temporal modelling. (C) Spatial transcriptomics has widened the horizon of cell–cell interaction studies. Both targeted and untargeted are being used to spatially resolve the transcriptome of several diseases at lower and higher resolution, respectively. (D) The full spectrum of disease heterogeneity can be unravelled by integrating several omics techniques, like genomics, transcriptomics, proteomics, epigenomics and metabolomics. This even though faces the disadvantage of technical difficulties of data integration, could be used with the help of deep‐learning techniques, and subsequently could revolutionise disease understanding. This figure is created in BioRender. Luo, Y. (2026) https://BioRender.com/e18pqmu.

Recent advancements in single‐cell technologies have expanded RNA transcriptomic profiling to include methods for single‐cell microRNA and single‐cell long non‐coding RNA.^301^ Techniques such as single‐cell metabolomics (sc‐metabolomics), have been proposed for the chemical content cataloguing of individual cells, or even organelles.^302^ single‐cell ribosomal profiling through scRibo‐seq offers the opportunity to examine translation at the single‐cell level. Integrated with a machine learning approach, this method achieves single codon resolution. 3D genome architectures in single cells can be detected using techniques like scSPRITE and Higashi (scHi‐C).^303^, ^304^ Overall, multi‐omics is a technique that can revolutionise the field of pharmaceutical science. But the high dimensionality and complexity of the data that is to be integrated pose immense challenges computationally and statistically. This could be subdued by generating, processing, reporting and evaluating the multi‐omics data with rigor and transparency in order to corroborate the high quality and validity of the data produced which requires scientists to take best practices and guidelines for multi‐omics data generation and validation (Figure 4).

## 12 CONCLUSION

The unprecedented sensitivity and granularity offered by scRNA‐seq technology have significantly advanced biomedical research, providing groundbreaking mechanistic insights into the biological diversity and cellular heterogeneity of underlying human diseases. Despite being only a decade old, scRNA‐seq techniques have evolved rapidly and are increasingly integrated with other single‐cell multi‐omics approaches and CRISPR screens at single‐cell resolution to enhance our understanding of human pathology. Furthermore, advancements in single‐cell spatial technologies have enabled the study of complex cellular interactions and distinct cellular niches within the tissues under investigation. This is also being used to complement the knowledge obtained from cellular interaction prediction obtained using scRNA‐seq data. Moreover, spatial transcriptomics are now being compiled with digital pathology and deep‐learning techniques to unravel the complex biology of diseases. Overall, the surge in multi‐omics studies holds promise for a comprehensive characterisation of gene regulatory processes, functions, molecules and interactions in both healthy and diseased tissues. However, integrating several multi‐omics data requires advanced computational expertise, which currently poses a significant challenge. The integration of AI and machine learning algorithms into big data analysis offers hope for overcoming these hurdles, potentially allowing scRNA‐seq and multi‐omics approaches to bridge the gap in our understanding of complex biological systems and help in crossing the ‘valley of death’.

## AUTHOR CONTRIBUTIONS

Conceptualisation: Yonglun Luo, Funding Acquisition: Yonglun Luo, Resources: Lin Lin and Yonglun Luo, Supervision, Lin Lin and Yonglun Luo, Visualisation: Aisha Shigna Nadukkandy and Sowmiya Kalaiselvan, Writing – Original Draft: Aisha Shigna Nadukkandy and Sowmiya Kalaiselvan, Writing – review and editing: Aisha Shigna Nadukkandy, Sowmiya Kalaiselvan, Lin Lin and Yonglun Luo.

## CONFLICT OF INTEREST STATEMENT

The authors declare no conflicts of interest.

## ETHICS STATEMENT

Not relevant for this study.

## Data Availability

Data sharing not applicable to this article as no datasets were generated or analysed during the current study.
